# A multistep procedure to prepare pre-vascularized cardiac tissue constructs using adult stem sells, dynamic cell cultures, and porous scaffolds

**DOI:** 10.3389/fphys.2014.00210

**Published:** 2014-06-03

**Authors:** Stefania Pagliari, Annalisa Tirella, Arti Ahluwalia, Sjoerd Duim, Marie-Josè Goumans, Takao Aoyagi, Giancarlo Forte

**Affiliations:** ^1^Biomaterials Unit, International Center for Materials Nanoarchitectonics, National Institute for Materials ScienceTsukuba, Japan; ^2^International Clinical Research Center, Integrated Center of Cellular Therapy and Regenerative Medicine, St. Anne's University HospitalBrno, Czech Republic; ^3^Interdepartmental Research Center “E. Piaggio”, University of PisaItaly; ^4^Institute of Clinical Physiology, National Research Council (CNR)Pisa, Italy; ^5^Department of Molecular Cell Biology, Leiden University Medical CenterLeiden, Netherlands

**Keywords:** cardiac tissue engineering, adult stem cells, vascularized three-dimensional (3D) scaffolds, dynamic culture, patient-derived stem cells

## Abstract

The vascularization of tissue engineered products represents a key issue in regenerative medicine which needs to be addressed before the translation of these protocols to the bedside can be foreseen. Here we propose a multistep procedure to prepare pre-vascularized three-dimensional (3D) cardiac bio-substitutes using dynamic cell cultures and highly porous biocompatible gelatin scaffolds. The strategy adopted exploits the peculiar differentiation potential of two distinct subsets of adult stem cells to obtain human vascularized 3D cardiac tissues. In the first step of the procedure, human mesenchymal stem cells (hMSCs) are seeded onto gelatin scaffolds to provide interconnected vessel-like structures, while human cardiomyocyte progenitor cells (hCMPCs) are stimulated *in vitro* to obtain their commitment toward the cardiac phenotype. The use of a modular bioreactor allows the perfusion of the whole scaffold, providing superior performance in terms of cardiac tissue maturation and cell survival. Both the cell culture on natural-derived polymers and the continuous medium perfusion of the scaffold led to the formation of a densely packaged proto-tissue composed of vascular-like and cardiac-like cells, which might complete maturation process and interconnect with native tissue upon *in vivo* implantation. In conclusion, the data obtained through the approach here proposed highlight the importance to provide stem cells with complementary signals *in vitro* able to resemble the complexity of cardiac microenvironment.

## Introduction

The successful regeneration of injured areas of the myocardium by tissue-engineered constructs relies on the long time viability and persistence of the bio-substitute *in vivo*, given the harsh conditions cells in the infarcted milieu are exposed to. Previous investigations revealed the sudden disappearance of cells administered by injection—systemically or locally—independent of cell type. This negative outcome has been ascribed to the low retention and high mortality of cells in the hypoxic environment characterized by an inflammatory response and the lack of local blood supply (Gnecchi et al., 2008; Menasche, 2011). The issue of promoting ischemic area vascularization has been lately addressed by cardiac tissue engineers through different approaches: (i) the administration of pro-angiogenic factors supplied by direct injection or through drug-releasing carriers (Sato et al., 2001; Chiu and Radisic, 2010; Singh et al., 2012); (ii) the infusion of endothelial progenitors (EPCs) or mature endothelial cells (ECs; Lian et al., 2008); and (iii) the pre-vascularization of the tissue constructs produced *in vitro* before implantation (Caspi et al., 2007; Dvir et al., 2009). Although the first two strategies are potentially interesting in a therapeutic perspective, they rely on the *in situ* generation and organization of vascular structures which depend either on the bioavailability of beneficial molecules or on the growth and differentiation capacity of vascular cells or their progenitors (Lovett et al., 2009). The early clinical trials in which growth factors or cells were delivered to the injured heart yielded disappointing results in terms of improvement of cardiac function (Urbich et al., 2005; Dubois et al., 2010; Simón-Yarza et al., 2012). The pre-vascularization of cardiac patches is also appropriate for providing a capillary network to support cells in the inner core of the implant, while biocompatible substrates are deemed to contribute to the improvement of retention and engraftment of the transplanted cardiac tissue (Terrovitis et al., 2010; Segers and Lee, 2011). The advantage of the pre-vascularization of thick muscle constructs was underlined by the demonstration that co-cultures including skeletal myoblasts, endothelial cells (or their progenitors) and embryonic fibroblasts on biocompatible porous scaffolds can enhance the overall survival and functionality of the constructs *in vivo* (Levenberg et al., 2005). Moreover, the adoption of scaffolds displaying an interconnected porosity itself could foster host vascular cell recruitment, with the possibility of vessels branching throughout the core of the construct. Alternatively, scaffoldless thick cardiac constructs were provided with a vascular bed (Sekine et al., 2013), or with microchannels (Sakaguchi et al., 2013) to favor vessel ingrowth, although biocompatible supports improve the handling of the grafts and can provide cells with appropriate bio-mechanical signals to better induce tissue regeneration and repair. In this context, the use of porous gelatin scaffolds represents a suitable tool for cardiac tissue engineering application (Sakai et al., 2001; Akhyari et al., 2002). In fact, gelatin is a cheap polymer derived from collagen denaturation and hydrolysis, and, due to its natural origin, it displays excellent cell adhesion property (Wu et al., 2011). It also features high biocompatibility, low immunogenicity, and biodegradability (Xing et al., 2014). In addition, gelatin sponges have been proven effective in inducing angiogenesis (Dreesmann et al., 2007) and their porous structure can favor the vascularization of the construct by supporting the diffusion of cells and nutrients within its core area. Its mechanical properties can be easily adjusted to match those encountered in living tissues.

The use of autologous stem cells has been proposed for various cell therapy applications as a mean to avoid the immune rejection issues raised by allogeneic or xenogeneic derivatives and the ethical concerns due to the use of embryonic material. Human bone marrow-derived mesenchymal stem cells (hMSCs) are an excellent candidate for regenerative medicine applications due to their autologous origin, their immunomodulatory properties and relative safety in clinical practice (Lalu et al., 2012). The *in vitro* multilineage differentiation potential of mesodermal progenitors has been proven in a number of studies (Pittenger et al., 1999; Muraglia et al., 2000) and their ability to express endothelial markers upon growth factor stimulation (Oswald et al., 2004; Jazayeri et al., 2008; Portalska et al., 2012) and response to bio-mechanical stimulation (stretching, shear stress, substrate mechanical properties tuning; Lozito et al., 2009; Bai et al., 2010) has been shown. More importantly, the benefits of MSC-based therapy have mainly been ascribed to their ability to generate endothelial cells and exert pro-angiogenic and cardioprotective effects by paracrine mechanisms rather than to direct the generation of new contractile cells (Gnecchi et al., 2008; Meyer et al., 2009; Wöhrle et al., 2010; Loffredo et al., 2011).

Among the adult stem cell subsets so far proposed for cardiac muscle repair, resident cardiac stem/progenitor cells (CSCs or CPCs) were shown to retain the ability to differentiate into all the cardiac tissue cell types (Beltrami et al., 2003; Forte et al., 2011) and favor cardiac healing by direct production of contractile cells *in vivo* (Smits et al., 2009a,b).

By taking advantage of the peculiar differentiation potential of hMSCs and human cardiomyocyte progenitor cells (hCMPCs), in the present investigation we propose a multistep procedure to obtain human pre-vascularized three-dimensional (3D) cardiac bio-substitutes based on highly porous gelatin scaffolds displaying the stiffness of cardiac tissue. Given the thickness and the dimensions of the bio-construct, a modular dynamic culture system has been used to guarantee scaffold perfusion and promote cell colonization of the inner layers.

Although being here tested exclusively *in vitro*, the present method allows for the formation of a 3D cardiac construct based on a physiological environment given by the gelatin scaffold and featuring the use of autologous stem cells with unique potential.

## Materials and methods

### Preparation of porous gelatin scaffolds

All materials used (unless specified) were purchased from Sigma-Aldrich (Italy). A 5% w/v gelatin solution was prepared by dissolving gelatin (Type A, G1890, 300 bloom strength) in deionized water. Porous gelatin scaffolds were prepared with a multi-step procedure. The solution was stirred for 1 h at 50°C, allowing complete dissolution, then casted in cylindrical shaped mold and physically gelled at room temperature. Samples were kept at 4°C for 1 h, and then at −20°C overnight. Gelatin samples were then freeze-dried (−50°C, 150 mBar) to obtain a porous structure as described elsewhere (Lien et al., 2009). Samples were swollen in deionized water and then cross-linked by immersion in a 10 mM glutaraldehyde (GTA) solution in 40% v/v ethanol/deionized water. The crosslinking reaction was controlled by keeping the glutaraldehyde/gelatin ratio (defined as molar concentration of GTA versus gelatin weight) constant. The scaffolds were immersed in GTA solution at 4°C for 48 h, until the cross-linking reaction occurred. Therefore, the samples were immersed in 0.1 M glycine solution in deionized water for 2 h at room temperature in order to stop any further cross-linking reaction (and remove any excess of GTA). Phosphate buffered solution (PBS) was sequentially used to rinse samples. Samples were kept at −20°C overnight, and finally freeze-dried (−50°C, 150 mBar) until all water content was removed. Samples were then stored at room temperature and sterilized with gas plasma before using.

### Scaffold mechano-architectural properties: swelling, porosity, and stiffness

Water absorption capability, porosity and stiffness of the porous gelatin scaffolds were evaluated using the procedure described elsewhere (Spinelli et al., 2012). Swelling ratio (*Q*) was calculated from the ratio of the weight of a dry (*W*_0_) and a completely swollen (*W*_eq_) sample (Brannon-Peppas and Peppas, 1990) returning the amount of adsorbed water. For the measurements, cryogel were swollen in deionized water at room temperature and weighted (*W*_i_) at different time points until a swelling equilibrium was reached. A precision microbalance (AE240, Mettler, Italy) was used: in case of wet samples, blotting paper was used to remove the water in excess. Porosity was indirectly evaluated by the imbibition method (Mwangi and Ofner, 2004; Martucci et al., 2006), while pore size was directly measured by processing both Scanning Electron Microscopy (SEM) and optical microscopy acquisition of sample sections with ImageJ (Abramoff et al., 2004). Sample stiffness was measured by compressive mechanical tests. Prior to the tests, samples were completely swollen in deionized water, compressive tests were then performed using a Zwick-Roell Z005 Instron twin column-testing machine (Zwick Testing Machines Ltd., UK). Samples were compressed up to 5% of their initial length using a 0.01 mm· *s*^−1^ strain rate; tests were performed with the samples partially immersed in water to preserve their hydration. Data were then post-processed and stress-strain curves were obtained. Samples stiffness was evaluated within 1% strain (first linear zone) of the stress-strain curve.

### Bioreactor working conditions

A computational fluid-dynamic (CFD) analysis of the modular chamber bioreactor with a porous scaffold was performed, assessing the perfusion and oxygenation of the millimeter sized scaffold. The analysis was performed using Brinkman and Incompressible Navier-Stokes equations were combined with reaction and diffusion equations using Comsol Multiphysics (COMSOL AB, Stockholm, Sweden). The system was then modeled with a porous domain representing the scaffold (porous section of 2 × 12 mm in size, with permeability of 1.68 × 10^−10^ m^2^ and 90% porosity) placed on the bottom of the bioreactor perfusion chamber, which is represented by a fluid domain. A preliminary analysis was performed in order to verify scaffold oxygenation as function of bioreactor flow rate (Supplementary Figure 1A). Chosen an inner flow rate to a value of 200 μ L/min, fluid flow inside the perfusion chamber and scaffold's perfusion was analyzed (Supplementary Figure 1B).

### Cell culture

hMSCs were purchased from Lonza and cultured in MSCGM™ BulletKit™ (hMSC basal medium, Lonza Japan Ltd, Tokyo, Japan). The cells were expanded to the desired number and used between passages 3 and 7. For the endothelial differentiation in 2D conditions, hMSCs were seeded onto tissue culture polystyrene plates (TCPS) and cultured for 7 days in endothelial differentiation medium [(EDM: hMSC basal medium supplemented with 50 ng/ml VEGF165 (R&D systems)]. hCMPCs were isolated from healthy donor atrial biopsies, cultured in basal medium (M199/EGM-2 (Lonza) (3:1) 10% FBS (Equitech-Bio Inc., Kerrville, Texas, USA), 1% MEM nonessential amino acids (from 50× stock; Life Technologies Gaithersburg, Maryland, USA) and 2% penicillin/streptomycin (from 50× stock; Invitrogen, Carlsbad, California, USA) and differentiated in cardiac differentiation medium (CDM: IMDM/Hams F12 (1:1, Life Technologies) with L-glutamine (Life Technologies), 2% horse serum (Life Tchnologies), nonessential amino acids, insulin–transferrin–selenium (Invitrogen), penicillin/streptomycin) as previously described (Smits et al., 2009a,b). Briefly, minced auricle were digested in collagenase A (1 mg/ml) for 2 h at 37°C while stirring. Afterwards, the solution was filtered through 40-μm cell strainer and centrifuged at 300 g for 5 min at RT. Then, the pellet was resuspended in cold buffer containing EDTA (2 mM) and 2% FBS and filtered through 40-μm cell strainer. The filtered cell population was subjected to magnetic cell sorting (Miltenyi Biotec, Sunnyvale, CA, USA) using Sca-1-coupled beads, following the manufacturer's protocol, in order to isolate Sca-1-like + progenitor cells. The cells were cultured on 0.1% gelatin- (Sigma-Aldrich, St Louis, Missouri, USA) coated plates in basal medium. For cardiac differentiation, cells were grown in differentiation medium supplemented with 5 μM 5-azacytidine (Wako Pure Chemical Industries, Ltd, Osaka, Japan) for 72 h. After induction, the medium was replaced with differentiation medium supplemented with 10^−4^ M ascorbic acid (Wako) and TGF-β 1 (1 ng/ml, PeproTech, Rocky Hill, NJ, USA) and changed every 3 days for up to 3 weeks. Human umbilical vein endothelial cells (HUVEC) were purchased from Lonza, grown in EGM-2 bullet kit media and used between passage 2 and 4. The media were replenished every other day.

### Generation of TNT-GFP hCMPCs

The pGreenZeo lentiviral expression vector carrying the full-length copGFP gene under the control of cardiac Troponin T type 2 (TNNT2) promoter was purchased from SBI (System Biosciences Inc. Mountain View, CA, USA) and delivered into mammalian cells according to the manufacturer's instructions. Briefly, hCMPCs were transduced at an approximate MOI of 20 and incubated at 37°C overnight. The following day, half of the culture medium was replaced with fresh medium and 48 h after the transduction, the medium was discarded and fresh medium added. The cells (hereafter referred as cTNT-GFP hCMPCs) were cultured on TCPS or gelatin scaffolds in the presence of basal medium or CDM.

### Cell seeding and 3D distribution in gelatin scaffolds

Prior to cell seeding, gelatin scaffolds were swollen in cell culture medium in Ultra-low Attachment 24-well plates (Corning® Incorporated) for 24 h. The cells (2.0 × 10^5^) were directly seeded on top of each scaffold in a small volume (100 μl) of appropriate medium and allowed to adhere for 60 min before adding the rest of medium. Then, the scaffolds were placed in the incubator for 12 h and transferred in another low-adhesion well. Cardiac TNT-GFP hCMPCs were cultured into scaffolds for 7 days in complete hCMPCs basal medium or CDM, while hMSCs-seeded scaffolds were cultured for 4 days in a mixture of Matrigel™/hMSCs basal medium (1:10) with or without VEGF (50 ng/mL) before being processed or co-cultured with pre-conditioned cardiac progenitors. The formation of vascular-like structures on gelatin scaffolds was followed by labeling hMSCs with the membrane-permeable live-cell labeling dye Calcein AM (Invitrogen) after 4 days of culture in EDM. Briefly, Calcein AM was added to hMSC-seeded scaffolds at a final concentration of 2 μ M for 30 min at 37°C and 5% CO_2_ in the dark. Five scaffolds have been used for any single experiment. The experiments have been performed independently three times.

### Dynamic culture

**Figure 1:**
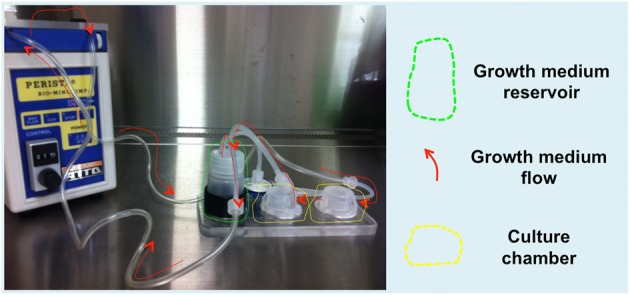
**QuasiVivo Bioreactor (QV500)**. The bioreactor is composed of a peristaltic pump keeping a constant medium flow rate (200 μl/min) from the growth medium reservoir to two culture chambers connected in series, thus allowing scaffold inner layer perfusion.

A commercial modular chamber bioreactor (Quasi-Vivo®, QV500, Kirkstall, United Kingdom) was used to perfuse the scaffolds. The main feature of this system is the ability to apply high flow rates and provide high nutrient turnover to cells without imposing high shear stress or turbulent flow (Mazzei et al., 2010). Pre-seeded scaffolds were gently transferred into the bioreactor chamber, and then the chamber was filled with cell culture media prior to perfusion. Two Quasi-Vivo® chambers and one mixing chamber (growth medium reservoir) were then connected in series to a peristaltic pump (High Performance Perista BIO-MINI PUMP, ATTO) giving a final volume of cell culture media of about 21 mL. The use of the mixing chamber guarantees medium oxygenation (Figure 1). To establish the optimal flow rate, the gelatin porous scaffold was included in the fluid-dynamic and oxygen transport model previously described (Mazzei et al., 2010), giving consistent results (Supplementary Figure 1). Cardiac TNT-GFP hCMPCs grown for 7 days onto gelatin scaffolds in the bioreactor were used to study the effect of dynamic culture conditions on cell growth, differentiation potential and gene expression profile. The medium was replenished every third day. For the generation of vascularized cardiac grafts, scaffolds were soaked for 12 h in diluted Matrigel™ (1:10 in hMSC basal medium) and switched to a new low-adhesion plate in EDM. The pre-conditioned scaffold was loaded with 2.0 × 10^5^ hMSCs and kept for 4 days in the incubator at 37°C, 5% CO_2_. After a 2 week pre-conditioning passage in CDM on TCPS, 2.0 × 10^5^ cTNT-GFP hCMPCs were seeded onto the vascularized scaffold and allowed to attach for 24 h. Thence, the cell-loaded scaffolds were carefully moved to Quasi-vivo bioreactor chambers and perfused with CDM at 200 μl/min (the rate suggested by the computational model) for 7 days. Cells grown on TCPS and 3D scaffolds under static conditions were used as controls. Five scaffolds have been used for any single experiment. The experiments have been performed independently three times.

### Real-time quantitative PCR analysis

Total RNA was extracted by TRIZOL® Reagent according to the manufacturer's instructions (Invitrogen). One microgram of RNA for each sample, measured by NanoDrop 2000 (Thermo Scientific) and assessed on ReadyAgarose Precast Gel (Bio-Rad), was retro-transcribed using RT^2^ First Strand Kit including DNAse treatment to remove genomic DNA (SA Biosciences Corp., USA). The resulting cDNA was diluted 1:10 in DNase/RNase-free water and the expression profile of genes involved in different pathways analyzed by the following RT^2^ Profiler™ PCR Arrays (SA Biosciences Corp.): (i) RT^2^ Profiler™ PCR Array Human Angiogenesis (PAHS-024Z); (ii) RT^2^ Profiler™ PCR Array Human Extracellular Matrix & Adhesion Molecules (PAHS-013); (iii) qBiomarker iPSC PCR Array Cardiomyocytes differentiation (iPHS102); (for a comprehensive list of genes included in these arrays please refer to Supplementary Table 1). Real-time PCR was performed on ABI 7500 Real-Time PCR System (AB Applied Biosystems, Foster City, CA) using RT^2^ SYBR Green/ROX qPCR Master Mix (SA Biosciences Corp.) and the following cycling parameters: 1 cycle at 95°C for 10 min; 40 cycles at 95°C for 15 s, and 60°C for 1 min. Data was analyzed by the ΔΔCt method with the PCR Array Data Analysis Web Portal (http://www.SABiosciences.com/pcrarraydataanalysis.php). The graphs show the mean values of obtained fold changes by analyzing independently two samples per each experimental condition.

### Statistical analysis

Data are represented as mean ± SD. Student's *T*-test was used to analyze the data. Comparisons between different conditions were considered statistically significant for *P* < 0.05.

For a more extensive description of the Materials and Methods used, please refer to Supplementary Information.

## Results

### Modeling the multi-dimensional *in vitro* system through gelatin scaffolds displaying cardiac-like mechanical properties

**Figure 2:**
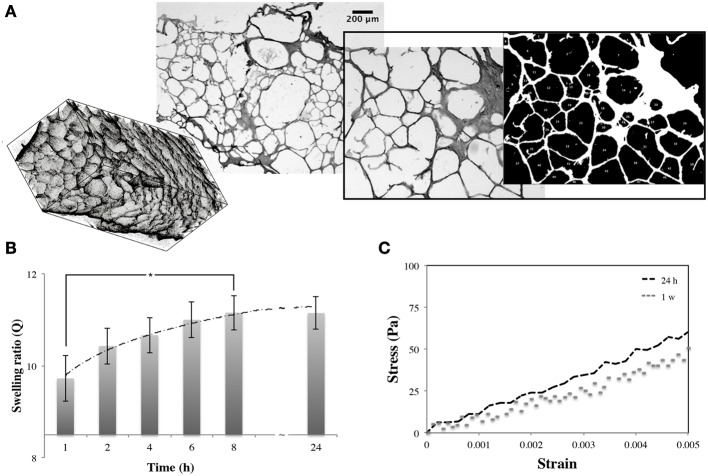
**The porous gelatin scaffold**. Three-dimensional rendering of scaffold, brightfield images of scaffold section and pore dimension analysis **(A)**. Modifications of swelling ratio of scaffold over 24 h in cell culture media **(B)**. Stress-strain plot of gelatin samples **(C)** obtained after 24 h and 1 week in culture medium (37°C, 5% CO_2_). ^*^P < 0.05.

Three dimensional gelatin scaffolds were characterized for their structure, water affinity, and stiffness. The analysis of the sections showed a distinctive bimodal pore size distribution. Although the average pore diameter ranged between 50–200 μm (with a few having a maximum size of 500 μm), the smaller pores were approximately double the size of the bigger pores (diameters of 61.62 ± 24.01 and 140.38 ± 19.51 μm, respectively; Figure 2A). Swelling tests were performed to assess the scaffold capacity to retain water. The scaffolds were monitored over 24 h of immersion in cell culture media, showing a complete rehydration after 8 h with a swelling ratio of about 10.92 ± 0.32 (Figure 2B). These data were also used to measure scaffold porosity, which was found to be approximately 90% (also confirmed in Spinelli et al., 2012). Finally, compressive mechanical tests were also performed on swollen samples (after 24 h in cell culture media), and on samples kept immersed in cell culture media and in the incubator for 1 week. Scaffold stiffness (evaluated within the first linear region of stress-strain plot, Figure 2C) was 10.5 ± 1.4 kPa, which was stable in time. After scaffold characterization and assessment of mechano-architectural properties, attention was dedicated to define the most suitable working conditions of the bioreactors to guarantee a homogeneous scaffold oxygenation in the bioreactor. Specifically, literature values of hypoxic limits (1%) (Cipolleschi et al., 1993) and oxygen consumption of stem cells (*K*_m_ = 0.001 mM, *V*_max_ = 1 pm/min/10^4^ cells) (Varum et al., 2011) were used to estimate the minimum oxygen concentration at the base of the scaffold as a function of flow rate and cell density, while the Wang and Tarbell approximation was used to calculate the shear stress at the pore walls. Results obtained from the computational models (reported in Supplementary Information) evidenced that—by using a flow rate of 200 μL/min—an oxygen concentration of 0.15 mM at the base of the scaffold (14%) and a shear stress of 1.10^−6^ Pa in the pores are obtained (Wang and Tarbell, 1995; Boschetti et al., 2006). The use of lower flow rates in the bioreactor may compromise oxygen supply rates to cells, especially as they proliferate, while higher flow rates may cause shear-related damage (Mazzei et al., 2010). Bioreactor parameters were set according to these results for all the following experiments.

### 3D culture induces a general remodeling in the expression of genes involved in cell-matrix interaction

**Figure 3:**
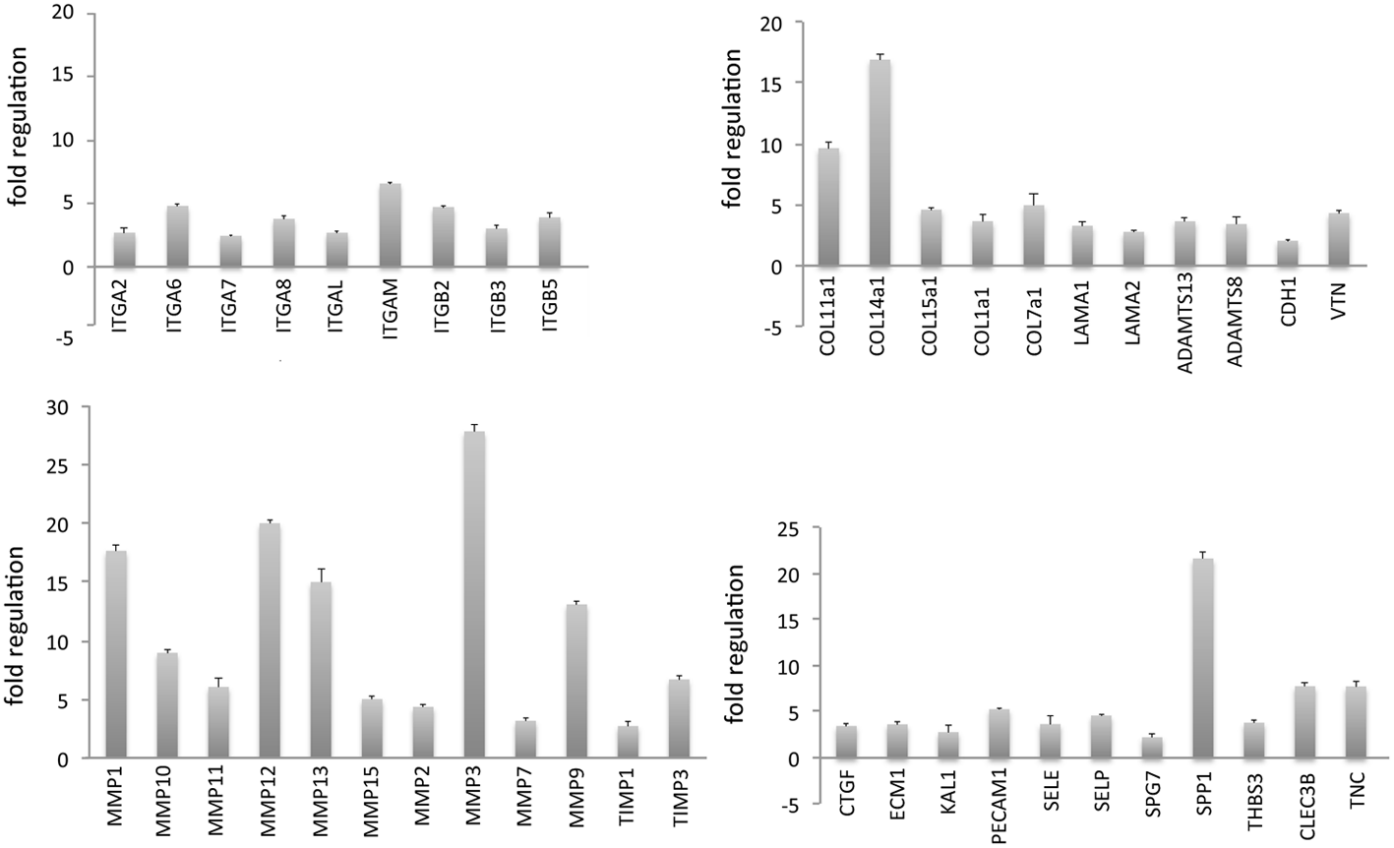
**Three-dimensional (3D) stem cell culture induces a significant remodeling in the expression of genes involved in cell adhesion and ECM deposition**. Human cardiac progenitor cells (hCMPCs) were grown for 1 week on 3D gelatin scaffolds and the expression of 73 genes involved in cell-matrix interaction resulted upregulated, as evidenced by real time PCR (for a complete list of genes analyzed please refer to Supplementary Table1).

Establishing a physiologically relevant system requires the development of 3D culture conditions resembling the *in vivo* microenvironment (Pampaloni et al., 2007). As reported above, the scaffolds here used were designed to have mechanical properties similar to those of cardiac tissue (Engler et al., 2007). Preliminary experiments demonstrated that culturing human hCMPCs on 3D gelatin scaffolds for 7 days induced a clear upregulation in the expression of genes (fold change ≥2, as compared to cells grown on TCPS) coding for proteins involved in cell-matrix remodeling (collagens (COL), laminins (LAMA), metalloproteinases (MMP) and their inhibitors (TIMP) and cell-cell adhesion (integrins) (Figure 3, for a complete list of the genes analyzed see Supplementary Table 1) and testified the actual activity of the cells in remodeling the scaffold.

### 3D porous gelatin scaffolds represent a permissive environment for stem cell cardiac and endothelial commitment

**Figure 4:**
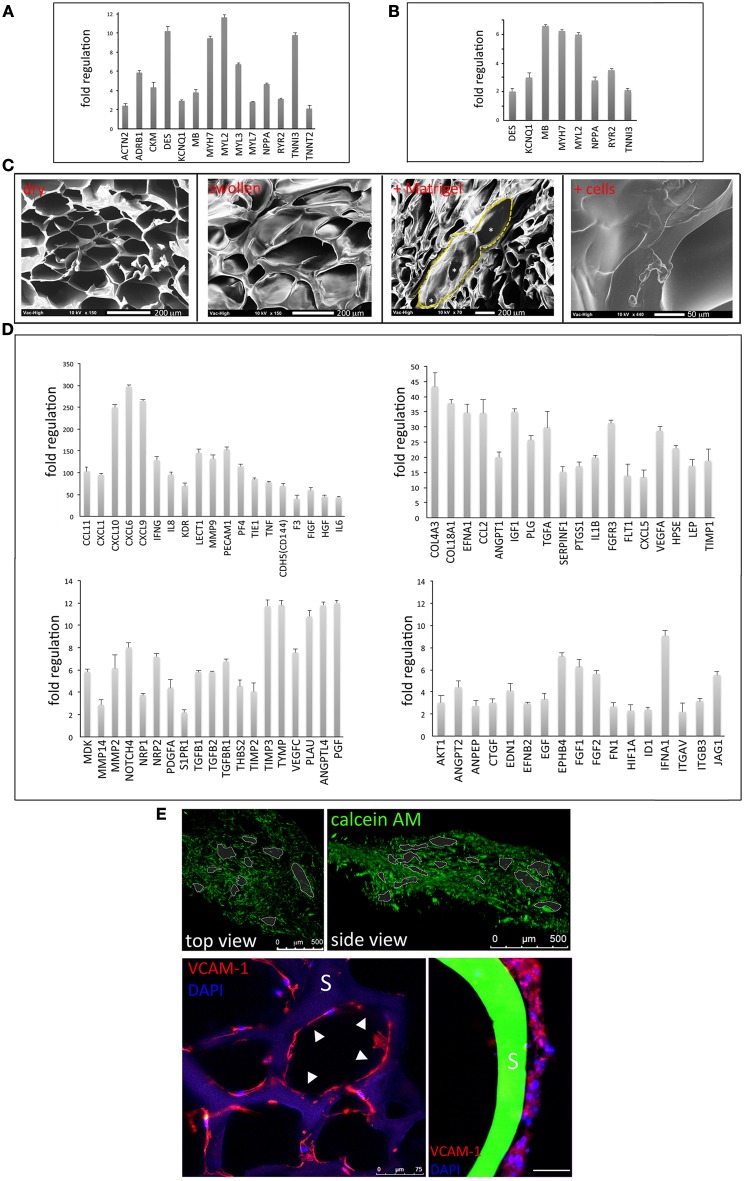
**Three-dimensional (3D) gelatin scaffolds support multilineage stem cell commitment**. After 2 weeks of cardiac pre-commitment on TCPS, human cardiac progenitors (hCMPCs), seeded on 3D gelatin scaffolds and stimulated with cardiogenic differentiation medium (CDM) for further 1 week, up-regulate some cardiac-specific genes, as compared to 3D culture in basal medium **(A)**. 3D differentiation conditions show a higher performance **(B)** as compared to tissue culture polystyrene (TCPS). The interconnected porosity of the scaffold (as outlined by yellow dashed line and asterisks) is not modified by Matrigel™ treatment and can accommodate human cells **(C)**. Human mesenchymal stem cells (hMSCs) are differentiated to endothelial cells on 3D gelatin scaffolds when stimulated with endothelial differentiation medium (EDM) and low concentration of Matrigel™, as demonstrated by the upregulation of several vascular genes as analyzed by real time PCR **(D)**. Calcein AM staining shows the network of living cells spreading throughout the scaffold (**E**, upper panels). Scaffold (S) pores are covered by lining cells expressing VCAM-1 as highlighted by white arrowheads (**E**, lower panel). A magnification of a cord-like structure aligned along a pore is visible on the right; scale bar: 100 μm.

The hCMPC reporter cell line (cTNT-GFP hCMPCs) was driven toward a cardiac phenotype in both 2D (Supplementary Figure 2) and 3D conditions, when stimulated with CDM (Figure 4A). Cell commitment was confirmed by the up-regulation of a number of cardiac-specific genes like actinin alpha 2 (ACTN2), adrenoceptor beta 1 (ADRB1), desmin (DES), cardiac troponin T type 2 (TNNT2), cardiac troponin I type 1 (TNNI1), myosin light chain (MYL) 2, 3, 7, myosin heavy chain b (MYH7) in cardiac progenitors stimulated on the scaffold as compared to their respective control (3D culture in basal medium). Moreover, when compared to cells cultured in CDM on TCPS, hCMPCs grown onto gelatin scaffolds encountered the up-regulation of few cardiac genes, confirming a superior performance of 3D vs. 2D culture conditions (Figure 4B, for a complete list of the genes analyzed see Supplementary Table 1). On the other hand, the ability of hMSCs to acquire an endothelial phenotype (Supplementary Figure 3) was exploited to obtain endothelial cells from hMSCs under 3D culture conditions. The scaffolds were treated with a mixture of EDM and Matrigel™ for 24 h. SEM analysis showed that the interconnected porosity of the scaffold was not modified by Matrigel™ treatment (highlighted by yellow dotted line in Figure 4C) and that the successful adhesion of human cells was achieved (Figure 4C, right). Consequently, hMSCs grown on 3D gelatin scaffolds in EDM displayed the up-regulation of a wide range of angiogenic markers as compared to cells cultured on scaffolds in basal medium (Figure 4D, for a complete list of the genes analyzed see Supplementary Table 1). Confocal analysis of 3D scaffolds after 4 day hMSC culture demonstrated the establishment that cells loaded with Calcein AM or expressing endothelial marker VCAM-1 colonized the whole scaffold, while aligning around the pores (Figure 4E).

### Dynamic culture conditions provide superior performance in terms of cardiac committment of hCMPCs

**Figure 5:**
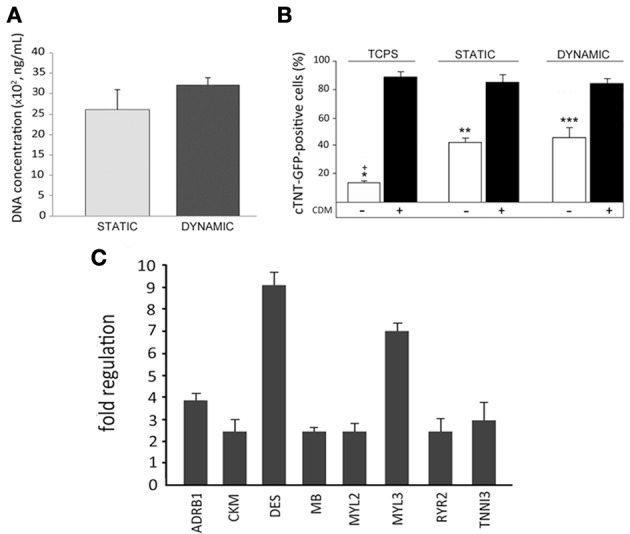
**Dynamic culture conditions induce a superior cardiac commitment in human cardiac progenitors (hCMPCs)**. Dynamic culture conditions provide a higher, although not significant, increase in scaffold cell colonization as compared to static conditions **(A)**. Percentage of differentiated cTNT-GFP CMPCs stimulated with cardiogenic differentiation medium (CDM) and cultured on TCPS and scaffolds, under dynamic, or static conditions, as evidenced by FACS analysis **(B)** (^*^, ^**^, ^***^P < 0.05 between hCMPC basal medium and CDM). Importantly, a significant commitment can be induced, equivalently, by 3D culture itself in both static and dynamic conditions compared to TCPS (^+^*P* < 0.05 between TCPS and 3D scaffolds). Nonetheless, quantitative PCR analysis demonstrates the up-regulation of few cardiac-specific genes in hCMPCs **(C)** cultured in dynamic conditions, as compared to its static control (for a complete list of genes analyzed please refer to Supplementary Table 1; among the genes analyzed, those not showing a consistent and significant difference in the expression as compared to their respective controls, are not shown).

The use of dynamic culture conditions using a bioreactor is thought to increase cell survival within a 3D structure by favoring inner core perfusion and catabolite removal (Cimetta et al., 2007; Burdick and Vunjak-Novakovic, 2009; Vozzi et al., 2011). Indeed, a slight although not significant increase in DNA content, indicative of cell proliferation, could be noticed 7 days after 3D cell culture in the bioreactor, as compared to static conditions (Figure 5A). Given the ability of human cardiac progenitor cells to proceed to cardiac maturation on 3D gelatin scaffolds, their capacity to activate the cardiac program when exposed to dynamic conditions in a modular bioreactor was assessed. For this experiment, cTNT-GFP hCMPCs were pre-committed in CDM for 2 weeks on TCPS. Afterwards, an aliquot of this cell population was seeded onto scaffolds and further stimulated for 1 week under static or dynamic conditions. The data confirmed that the commitment of cTNT-GFP hCMPCs could be achieved by CDM in dynamic conditions (Figure 5B) and that a minor although significant spontaneous commitment of hCMPCs was induced by 3D culture conditions *per se*, as also described in static culture (white columns). Although the dynamic culture conditions did not significantly modify the percentage of GFP-positive cells with respect to the static protocol, real time PCR array analysis demonstrated that dynamic stimulation—when combined with differentiation medium—could lead to the up-regulation of few cardiac-specific genes (Figure 5C, for a complete list of the genes analyzed see Supplementary Table 1).

### Generation of 3D pre-vascularized cardiac constructs by dynamic culture and porous scaffolds

**Figure 6:**
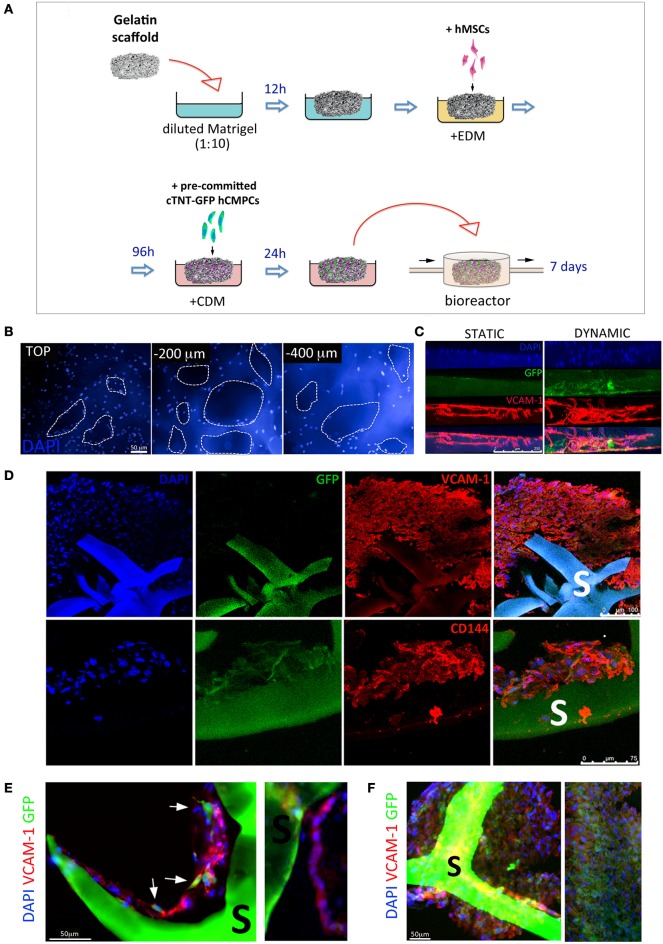
**Generation of a vascularized 3D cardiac construct by adult stem cells grown in dynamic conditions onto gelatin porous scaffolds**. Schematic illustration of the protocol used to produce vascularized 3D cardiac construct **(A)**. Gelatin porous sponges were dipped in diluted Matrigel™ and then switched to endothelial differentiation medium (EDM). Therefore, hMSCs were allowed to colonize the scaffold and differentiate toward the endothelial phenotype for 4 days. Cardiac TNT-GFP progenitor cells were pre-committed for 2 weeks with cardiac differentiation medium (CDM) on TCPS, and then cultured on vascularized scaffold for further 7 days in CDM and in a perfusive modular bioreactor. Human cells colonize the scaffold inner layers as shown by nuclei staining at different depths **(B)**. Side view of cellularized scaffolds cultured under static or dynamic conditions **(C)**; infiltration of GFP- (cardiomyocyte-like cells, green) and VCAM-1-positive cells (endothelial-like cells, red) into scaffold is improved by dynamic culture. Immunohistochemistry analysis of the colonized scaffolds shows the massive infiltration of VCAM-1, CD144 cells in the core of the construct. Higher magnification images **(D)** shows the VCAM-1-positive cells aligned in tube-like structures around the pores and contacting GFP-positive cells **(E,F)**. S, scaffold.

Given the positive effect of dynamic culture conditions on cardiac gene activation, we sought to obtain vascularized cardiac 3D tissues by co-culturing hMSCs-derived vascular cells and pre-differentiated hCMPCs in the bioreactor system for 7 days, as described in Figure 6A. Gelatin sponges were first seeded with hMSCs under endothelial differentiation conditions. In the meantime, cTNT-GFP hCMPCs seeded on TCPS were challenged with CDM for 2 weeks, seeded onto hMSCs-loaded scaffolds and allowed to adhere for 24 h before being exposed to continuous medium flow. The analysis of the co-cultured constructs after 1 week testified massive scaffold colonization by the cells (Figure 6B). The scaffold thickness was not significantly modified by medium perfusion (data not shown) that affected, instead, cell migration inside the scaffold. In fact, the side of the colonized scaffolds directly exposed to flow showed a more uniform and extensive distribution of vascular-like cells (in red) and hCMPC-derived cardiomyocytes (green) inside the scaffold, while the cells were mostly distributed on the scaffold surface in the static control (Figure 6C). The expression of VCAM-1 and CD144 endothelial markers confirmed the presence of endothelial-like cells, while GFP expression testified the persistence of a number of cardiac-like cells within the bio-construct (Figure 6D). Sections from the perfusion group showed VCAM-1-positive cells aligned forming tube-like structures around the pores and contacting GFP-positive cells (Figure 6E). The extensive cell distribution within the scaffold accounted for the formation of a densely packed multicellular tissue derived from the two different stem cell types used (Figure 6F).

## Discussion

Rebuilding functional portions of the myocardium requires the generation of bio-substitutes that best recapitulate the structure and function of the healthy myocardium, thus providing new cardiomyocytes with a functional vascular network, which may prevent or reduce pathological decline and improve cardiac function after injury (Simons and Ware, 2003; Kang et al., 2013). Exploiting cell sheet technology, our group and others successfully achieved the preparation of 3D cardiac tissues in the absence of scaffolds by different cell types having a cardiac significance (Haraguchi et al., 2006; Forte et al., 2011; Matsuura et al., 2012; Sekine et al., 2013). Nonetheless, the scaling up of solid engineered tissues to obtain a critical size substitute with therapeutical relevance is limited by the diffusion of oxygen, nutrients and waste products to and from the inner portion of the construct, impairing the survival of the newly formed tissue. In this context, the use of porous scaffolds to generate *in vitro* artificial bio-substitutes gives the opportunity to choose the shape and the size of the graft, while second-generation scaffolds are currently proposed as active templates which can be removed in a timely fashion compatible with tissue growth and exert an instructive role in the formation of the new tissue (Forte et al., 2013; Pagliari et al., 2013). In the present study, we propose a multistep procedure to prepare adult stem cell-derived pre-vascularized thick cardiac patches by using 3D porous gelatin scaffolds and dynamic culture conditions. The procedure here adopted is based on the peculiar differentiation potential of two different autologous adult cell subsets, mesenchymal, and cardiac resident stem cells. Human MSCs can be routinely obtained from patient bone marrow and display a minimal, if any, ability to give raise to cardiomyocytes (Gnecchi et al., 2005; Loffredo et al., 2011), while they are supposed to retain an intrinsic vascular potential (Oswald et al., 2004). In the present investigation, hMSCs were efficiently driven to acquire an endothelial phenotype by means of EDM in 3D culture conditions. In particular, when grown on 3D porous gelatin scaffold, hMSCs showed a robust vascular commitment as confirmed by the up-regulation of 73 genes involved in angiogenesis. Among these, angiopoietin 1 (ANGPT1), which has been shown to promote blood vessel maturation by regulating endothelial cell survival and the recruitment of mural cells (Thomas and Augustin, 2009), encountered a 20-fold increase, thus suggesting stability of the newly formed vascular network. At the same time, CPCs can be extracted from patient atrial biopsies and are known to possess the potential to generate new cardiomyocytes *in vitro* and *in vivo* (Beltrami et al., 2003; Goumans et al., 2007) making them an effective and safe cardiogenic cell type (Bolli et al., 2011; Makkar et al., 2012). In the present investigation, we explored the possibility to generate a 3D cardiac tissue *in vitro* by pre-committed hCMPCs. Although most of the attempts to regenerate damaged myocardium have used undifferentiated cells, concerns have been raised due to the poor differentiation capability of adult progenitors *in vivo*, together with the potential of pluripotent stem cells to give birth to undesired phenotypes. Recently, it has been suggested that the transplantation of pre-committed cells could improve the clinical outcome in cardiac patients (Heng et al., 2004; Mehta and Shim, 2013). For this reason, stem/progenitor cells were appropriately stimulated to undergo tissue specific pre-commitment before being used for all co-culture experiments. However, given that the mechanical properties of the gelatin scaffolds used here are similar to those of cardiac tissue, a spontaneous commitment of cardiac progenitor cells could be obtained through 3D culture *per se*, as demonstrated by the increased percentage of GFP-positive cells and similarly to what we and others previously demonstrated (Engler et al., 2006; Pagliari et al., 2011; Gaetani et al., 2012; Mosqueira et al., 2014). However, despite the fact that the gene expression profile analysis confirmed a moderate commitment of hCMPCs induced by the scaffold itself, biological factors were needed to improve this effect. In particular, when cells seeded on the scaffold were stimulated with CDM, we observed an increased expression of cardiac troponin genes (TNNT2, 3) along with the up-regulation of ryanodine receptor 2 (RYR2, involved in muscle excitation-contraction coupling, Bround et al., 2012) and natriuretic peptide precursor A (NPPA) genes. These data suggest that the combination of 3D porous gelatin and CDM represents a suitable combination to provide cells with biological and mechanical signals synergistically supporting the commitment of hCMCPs at a later stage. Nonetheless, neither complete sarcomeric structures nor any beating activity could be noticed in prompted cells. This evidence suggested that the system implemented represents a good tool to induce an efficient cardiac commitment and study the behavior of late cardiac progenitors, but this also implicates that more complex devices providing stretching and/or electrical stimulation are probably necessary to deliver more specific stimuli and improve the outcome in terms of cardiac differentiation. The activation of genes involved in matrix remodeling observed in 3D testified the ability of cells to actively interact with the natural support they grow on. In fact, while the up-regulation of Matrix metalloproteinase (MMP) genes and their regulators, tissue inhibitors of metalloproteinase (TIMP), is predictive of gelatin remodeling and breakdown, the activation of genes encoding for different collagens and laminins suggest the concomitant substitution of the artificial matrix with neo-formed tissue.

Bioreactors have been widely proposed to intensify oxygen and nutrient transport and increase the viability and function of thick cardiovascular tissue-engineered substitutes (Zimmermann et al., 2006; Cimetta et al., 2007; Burdick and Vunjak-Novakovic, 2009). In our experimental setting, dynamic culture conditions positively affected cardiac commitment, leading to a significant increase in GFP-expressing cells as compared to TCPS and independently of biological stimulation. Since the increase in GFP was not significantly different from that observed under 3D static culture conditions, it might be concluded that providing cells with continuous medium flow does not positively affect on cardiac progenitor differentiation potential on the scaffold. Nevertheless, the expression of genes recognized to be essential for the formation or regulation of the contractile apparatus was positively influenced when dynamic flow was applied to cells grown in a 3D environment. Hence, the use of a modular bioreactor providing continuous media turnover to the scaffold resulted in a superior performance in terms of cardiac gene activation. In the light of these promising results, we also expected that direct perfusion of cells could promote the formation of a vascularized cardiac patch. In line with previous descriptions (Maidhof et al., 2012), we observed more uniform and dense spatial cell distribution in the scaffold, with cells migrating toward the substrate inner core under dynamic conditions. Thus we established a method for sequential cell seeding: gelatin scaffolds were colonized with hMSCs and maintained in static conditions for 4 days in order to favor the endothelialization of the substrate, then scaffolds were loaded with pre-committed GFP-positive cardiac progenitors and cultured in perfusion bioreactor for 1 week in the presence of cardiogenic medium. As a result, a 3D cardiac proto-tissue composed of densely packed cardiomyocyte-like cells intertwined with vessel-like structures was obtained. Although being promising in terms of cell colonization and survival, these co-culture conditions did not yield the formation of functional contractile and vascular structures. Thus additional experiments will be necessary to provide cells with other cues *in vitro* to complete the organization of a proper vascularized cardiac tissue.

Nonetheless, irrespective of the stem cell subsets used, the procedure here described provides a novel platform for the preparation of complex 3D vascularized bio-constructs to be used for *in vitro* studies aiming at the understanding of stem cell behavior in a more physiological context. Pre-clinical animal studies addressing the long-term *in vivo* survival and the relevance of the constructs will be necessary before this technique may represent a suitable platform for future clinical applications.

### Conflict of interest statement

The authors declare that the research was conducted in the absence of any commercial or financial relationships that could be construed as a potential conflict of interest.
