# Supplementary material for: A multistep procedure to prepare pre-vascularized cardiac tissue constructs using adult stem sells, dynamic cell cultures, and porous scaffolds
Source: Front Physiol. 2014 Jun 3;5:210. doi: 10.3389/fphys.2014.00210 (PMC4042082; doi:10.3389/fphys.2014.00210)
Supplement: Supplementary file 1 [file DataSheet1.DOCX]

***Supplementary Material***

**A Multistep Procedure To Prepare Pre-Vascularized Cardiac Tissue Constructs Using Adult Stem Sells, Dynamic Cell Cultures And Porous Scaffolds**

Pagliari S^1^, Tirella A^2,3^, Ahluwalia A^2,3^, Duim S^4^, Goumans MJ^4^, Aoyagi T^1^, Forte G^1,5^.

^1^Biomaterials Unit, International Center for Materials Nanoarchitectonics (MANA), National Institute for Materials Science (NIMS), Tsukuba, Japan.

^2^Interdepartmental Research Center “E. Piaggio”, University of Pisa, (Italy)

^3^Institute of Clinical Physiology, National Research Council (CNR), Pisa, (Italy)

^4^Department of Molecular Cell Biology, Leiden University Medical Center, The Netherlands.

^5^International Clinical Research Center (ICRC), Integrated Center of Cellular Therapy and Regenerative Medicine, St. Anne´s University Hospital, Brno, Czech Republic

**Correspondence:**

Giancarlo Forte, PhD

International Clinical Research Center (ICRC),

Integrated Center of Cellular Therapy and Regenerative Medicine,

St. Anne´s University Hospital,

65691 Brno, Czech Republic

Email: [Giancarlo.forte@fnusa.cz](mailto:Giancarlo.forte@fnusa.cz)

Takao Aoyagi, PhD

Smart Biomaterials Group

International Center for Materials Nanoarchitectonics (MANA)

National Institute for Materials Science (NIMS)

1-1 Namiki

305-0051, Tsukuba

Ibaraki, Japan

Email: [Aoyagi.takao@nims.go.jp](mailto:Aoyagi.takao@nims.go.jp)

1. **Supplementary Data**

**Supplementary Materials and Methods**

**Preparation of porous gelatin scaffolds**

All materials used (unless specified) were purchased from Sigma-Aldrich (Italy). A 5% w/v gelatin solution was prepared by dissolving gelatin (Type A, G1890, 300 bloom strength) in deionized water. Porous gelatin scaffolds were prepared with a multi-step procedure. The solution was stirred for 1 hour at 50°C, allowing complete dissolution, then casted in cylindrical shaped mold and physically gelled at room temperature. Samples were kept at 4°C for 1 hour, and then at -20°C overnight. Gelatin samples were then freeze-dried (-50°C, 150 mBar) to obtain a porous structure as described elsewhere (Lien et al., 2009). Samples were swollen in deionized water and then cross-linked by immersion in a 10 mM glutaraldehyde (GTA) solution in 40% v/v ethanol/deionized water. The crosslinking reaction was controlled by keeping the glutaraldehyde/gelatin ratio (defined as molar concentration of GTA versus gelatin weight) constant. The scaffolds were immersed in GTA solution at 4°C for 48 hours, until the cross-linking reaction occurred. Therefore, the samples were immersed in 0.1 M glycine solution in deionized water for 2 hours at room temperature in order to stop any further cross-linking reaction (and remove any excess of GTA). Phosphate buffered solution (PBS) was sequentially used to rinse samples. Samples were kept at -20°C overnight, and finally freeze-dried (-50°C, 150 mBar) until all water content was removed. Samples were then stored at room temperature and sterilized with gas plasma before using.

**Scaffold mechano-architectural properties: swelling, porosity and stiffness**

Water absorption capability, porosity and stiffness of the porous gelatin scaffolds were evaluated using the procedure described elsewhere (Spinelli et al., 2012).

Swelling ratio (Q) was calculated from the ratio of the weight of a dry (W_0_) and a completely swollen (W_eq_) sample (Brannon-Peppas and Peppas, 1990) returning the amount of adsorbed water. For the measurements, cryogel were swollen in deionized water at room temperature and weighted (W_i_) at different time points until a swelling equilibrium was reached. A precision microbalance (AE240, Mettler, Italy) was used: in case of wet samples, blotting paper was used to remove the water in excess. Porosity was indirectly evaluated by the imbibition method (Martucci et al., 2006; Mwangi et al., 2004), while pore size was directly measured by processing both Scanning Electron Microscopy (SEM) and optical microscopy acquisition of sample sections with ImageJ (Abramoff et al., 2004).

Sample stiffness was measured by compressive mechanical tests. Prior to the tests, samples were completely swollen in deionized water, compressive tests were then performed using a Zwick-Roell Z005 Instron twin column-testing machine (Zwick Testing Machines Ltd., UK). Samples were compressed up to 5% of their initial length using a 0.01 mm·s^-1^ strain rate; tests were performed with the samples partially immersed in water to preserve their hydration. Data were then post-processed and stress-strain curves were obtained. Samples stiffness was evaluated within 1% strain (first linear zone) of the stress-strain curve.

**Bioreactor working conditions**

A computational fluid-dynamic (CFD) analysis of the modular chamber bioreactor with a porous scaffold was performed, assessing the perfusion and oxygenation of the millimeter sized scaffold. The analysis was performed using Brinkman and Incompressible Navier-Stokes equations were combined with reaction and diffusion equations using Comsol Multiphysics (COMSOL AB, Stockholm, Sweden). The system was then modeled with a porous domain representing the scaffold (porous section of 2 × 12 mm in size, with permeability of 1.68×10^-10^ m^2^ and 90% porosity) placed on the bottom of the bioreactor perfusion chamber, which is represented by a fluid domain. A preliminary analysis was performed in order to verify scaffold oxygenation as function of bioreactor flow rate (Supplementary Fig. S1a). Chosen an inner flow rate to a value of 200 μL/min, fluid flow inside the perfusion chamber and scaffold’s perfusion was analyzed (Supplementary Fig. S1b).

**Matrigel™ assay**

Tube formation assay was performed by seeding 4.0x10^4^ cells/ well in 96-well plates pre-coated with 50 μL of cold Growth Factor Reduced Matrigel™ basement membrane matrix, Phenol Red free (BD Biosciences). The assay was performed in the presence or absence of 50 ng/ml VEGF. The formation of tubes was observed using an inverted phase–contrast light microscope (Olympus IX71) after 6-hour incubation. Eight random fields per well were photographed (10x magnification). Forty-eight hours after seeding, the number and cumulative length of all capillary-like sprouts originating from the perimeter of an individual spheroid were measured, with 8 spheroids analyzed per experimental group in 4 independent experiments. The number and length of tube-like structures were measured by manual tracing of vessels by using ImageJ software (version 1.46; National Institutes of Health, Bethesda, MD).

**Fluorescence-activated cell sorting (FACS) analysis**

At different time-points, the percentage of TNT-GFP positive hCMPCs was assessed by FACSCalibur flow cytometer (BD Biosciences Franklin Lake, NJ, USA). A total of 10,000 events were scored. Data were acquired using Cell Quest software and the analysis done using WinMDI 2.9 software.

**Immunofluorescence**

The protocol for immunofluorescence has been described in a previous report (Forte et al., 2012). The endothelial differentiation of hMSCs was evaluated by antibodies directed against von Willebrand Factor (vWF, Abcam, Cambridge, UK), vascular cell adhesion molecule 1 (VCAM-1, BioLegend, San Diego, CA), CD144 or VE-cadherin (BD Bioscences), and by the uptake of endothelial specific acetylated low-density lipoprotein (LDL) labeled with 1,10-dioctadecyl-3,3,30,30-tetramethylindocarbocyanine (Dil-Ac-LDL, Life Technologies) in accordance with manufacturer’s instructions. Anti-cardiac troponin T-C (cTNT, Santa Cruz Biotechnology Inc., California, USA), -α-sarcomeric actinin (α-act, Sigma Aldrich) and –turboGFP (Evrogen, JSC, Russia) antibodies were used to label differentiated cardiac cells. Appropriate secondary antibodies (488-, 546-Alexa Fluor, Life Technologies) were applied. Nuclei were counterstained with 4–6-diamidino-2-phenylindole (DAPI; Sigma-Aldrich). The samples were mounted using ProLong Gold Antifade Reagent (LifeTechnologies) and imaged using a Leica SF5 laser scanning confocal microscope or Olympus IX-81 inverted microscope. All pictures are representative of at least 3 independent experiments.

**Scanning electron microscopy (SEM) analysis**

SEM analysis of gelatin scaffolds, prepared as previously reported (Forte et al., 2012), was performed using Hitachi S-4800 low-voltage scanning electron microscope. In particular samples were morphologically analysed in different state: i) dry, ii) swollen with medium, iii) swollen with diluted Matrigel and iv) seeded with cells.

**Analysis of DNA content (PicoGreen® assay)**

To quantify double-stranded DNA content within the samples obtained from the different experimental groups, dry scaffolds with equal weights and dimensions were selected and seeded with 2.0x10^5^ cTNT-GFP hCMPCs. The cells were allowed to grow for 1 week under static or dynamic conditions. The samples were then lysed in 300 μL proteinase k solution (1 mg/mL, Wako), 10mM Tris-HCl, 1mM EDTA, pH 7.5, 0.1% Triton X-100 for 30 min at 55°C. After centrifugation at 10000 x g, 10 min at room temperature, the supernatants were collected, diluted 1:10 in Tris-EDTA buffer and DNA content measured in triplicate in black 96-well plates by Quant-iT™ PicoGreen® dsDNA assay kit (Molecular Probes) according to the manufacturer’s instructions. The fluorescence of the DNA was measured at excitation and emission wavelengths of 485 nm and 530 nm respectively, using MTP-880 Lab microplate spectrofluorometer (Corona Electric, Ibaraki, Japan). The amount of DNA was calculated by interpolation from a standard curve prepared using lambda DNA (from 1 ng to 1μg).

1. **Supplementary Figures and Tables**

## Supplementary Tables

**Supplementary Table 1. Gene table of RT² Profiler™ PCR Arrays used.**

| **PCR Array Human Extracellular Matrix & Adhesion Molecules**  **( PAHS-013Z )** | | | | | | | | | |
| --- | --- | --- | --- | --- | --- | --- | --- | --- | --- |
| **Unigene** | | **GeneBank** | | **Symbol** | | **Description** | | **Gene Name** | |
| Hs.643357 | | NM_006988 | | ADAMTS1 | | ADAM metallopeptidase with thrombospondin type 1 motif, 1 | | C3-C5, METH1 | |
| Hs.131433 | | NM_139025 | | ADAMTS13 | | ADAM metallopeptidase with thrombospondin type 1 motif, 13 | | ADAM-TS13, ADAMTS-13, C9orf8, VWFCP, vWF-CP | |
| Hs.271605 | | NM_007037 | | ADAMTS8 | | ADAM metallopeptidase with thrombospondin type 1 motif, 8 | | ADAM-TS8, METH2 | |
| Hs.502328 | | NM_000610 | | CD44 | | CD44 molecule (Indian blood group) | | CDW44, CSPG8, ECMR-III, HCELL, HUTCH-I, IN, LHR, MC56, MDU2, MDU3, MIC4, Pgp1 | |
| Hs.461086 | | NM_004360 | | CDH1 | | Cadherin 1, type 1, E-cadherin (epithelial) | | Arc-1, CD324, CDHE, ECAD, LCAM, UVO | |
| Hs.476092 | | NM_003278 | | CLEC3B | | C-type lectin domain family 3, member B | | TN, TNA | |
| Hs.739161 | | NM_001843 | | CNTN1 | | Contactin 1 | | F3, GP135 | |
| Hs.523446 | | NM_080629 | | COL11A1 | | Collagen, type XI, alpha 1 | | CO11A1, COLL6, STL2 | |
| Hs.101302 | | NM_004370 | | COL12A1 | | Collagen, type XII, alpha 1 | | BA209D8.1, COL12A1L, DJ234P15.1 | |
| Hs.409662 | | NM_021110 | | COL14A1 | | Collagen, type XIV, alpha 1 | | UND | |
| Hs.409034 | | NM_001855 | | COL15A1 | | Collagen, type XV, alpha 1 | | - | |
| Hs.368921 | | NM_001856 | | COL16A1 | | Collagen, type XVI, alpha 1 | | 447AA | |
| Hs.681002 | | NM_000088 | | COL1A1 | | Collagen, type I, alpha 1 | | OI4 | |
| Hs.508716 | | NM_001846 | | COL4A2 | | Collagen, type IV, alpha 2 | | ICH, POREN2 | |
| Hs.210283 | | NM_000093 | | COL5A1 | | Collagen, type V, alpha 1 | | - | |
| Hs.474053 | | NM_001848 | | COL6A1 | | Collagen, type VI, alpha 1 | | OPLL | |
| Hs.420269 | | NM_001849 | | COL6A2 | | Collagen, type VI, alpha 2 | | PP3610 | |
| Hs.476218 | | NM_000094 | | COL7A1 | | Collagen, type VII, alpha 1 | | EBD1, EBDCT, EBR1 | |
| Hs.740613 | | NM_001850 | | COL8A1 | | Collagen, type VIII, alpha 1 | | C3orf7 | |
| Hs.410037 | | NM_001901 | | CTGF | | Connective tissue growth factor | | CCN2, HCS24, IGFBP8, NOV2 | |
| Hs.656653 | | NM_001903 | | CTNNA1 | | Catenin (cadherin-associated protein), alpha 1, 102kDa | | CAP102 | |
| Hs.476018 | | NM_001904 | | CTNNB1 | | Catenin (cadherin-associated protein), beta 1, 88kDa | | CTNNB, MRD19, armadillo | |
| Hs.166011 | | NM_001331 | | CTNND1 | | Catenin (cadherin-associated protein), delta 1 | | CAS, CTNND, P120CAS, P120CTN, p120, p120(CAS), p120(CTN) | |
| Hs.314543 | | NM_001332 | | CTNND2 | | Catenin (cadherin-associated protein), delta 2 (neural plakophilin-related arm-repeat protein) | | GT24, NPRAP | |
| Hs.81071 | | NM_004425 | | ECM1 | | Extracellular matrix protein 1 | | URBWD | |
| Hs.203717 | | NM_002026 | | FN1 | | Fibronectin 1 | | CIG, ED-B, FINC, FN, FNZ, GFND, GFND2, LETS, MSF | |
| Hs.57697 | | NM_001523 | | HAS1 | | Hyaluronan synthase 1 | | HAS | |
| Hs.643447 | | NM_000201 | | ICAM1 | | Intercellular adhesion molecule 1 | | BB2, CD54, P3.58 | |
| Hs.644352 | | NM_181501 | | ITGA1 | | Integrin, alpha 1 | | CD49a, VLA1 | |
| Hs.482077 | | NM_002203 | | ITGA2 | | Integrin, alpha 2 (CD49B, alpha 2 subunit of VLA-2 receptor) | | BR, CD49B, GPIa, HPA-5, VLA-2, VLAA2 | |
| Hs.265829 | | NM_002204 | | ITGA3 | | Integrin, alpha 3 (antigen CD49C, alpha 3 subunit of VLA-3 receptor) | | CD49C, GAP-B3, GAPB3, ILNEB, MSK18, VCA-2, VL3A, VLA3a | |
| Hs.440955 | | NM_000885 | | ITGA4 | | Integrin, alpha 4 (antigen CD49D, alpha 4 subunit of VLA-4 receptor) | | CD49D, IA4 | |
| Hs.505654 | | NM_002205 | | ITGA5 | | Integrin, alpha 5 (fibronectin receptor, alpha polypeptide) | | CD49e, FNRA, VLA5A | |
| Hs.133397 | | NM_000210 | | ITGA6 | | Integrin, alpha 6 | | CD49f, ITGA6B, VLA-6 | |
| Hs.524484 | | NM_002206 | | ITGA7 | | Integrin, alpha 7 | | - | |
| Hs.171311 | | NM_003638 | | ITGA8 | | Integrin, alpha 8 | | - | |
| Hs.174103 | | NM_002209 | | ITGAL | | Integrin, alpha L (antigen CD11A (p180), lymphocyte function-associated antigen 1; alpha polypeptide) | | CD11A, LFA-1, LFA1A | |
| Hs.172631 | | NM_000632 | | ITGAM | | Integrin, alpha M (complement component 3 receptor 3 subunit) | | CD11B, CR3A, MAC-1, MAC1A, MO1A, SLEB6 | |
| Hs.436873 | | NM_002210 | | ITGAV | | Integrin, alpha V (vitronectin receptor, alpha polypeptide, antigen CD51) | | CD51, MSK8, VNRA, VTNR | |
| Hs.643813 | | NM_002211 | | ITGB1 | | Integrin, beta 1 (fibronectin receptor, beta polypeptide, antigen CD29 includes MDF2, MSK12) | | CD29, FNRB, GPIIA, MDF2, MSK12, VLA-BETA, VLAB | |
| Hs.375957 | | NM_000211 | | ITGB2 | | Integrin, beta 2 (complement component 3 receptor 3 and 4 subunit) | | CD18, LAD, LCAMB, LFA-1, MAC-1, MF17, MFI7 | |
| Hs.218040 | | NM_000212 | | ITGB3 | | Integrin, beta 3 (platelet glycoprotein IIIa, antigen CD61) | | BDPLT16, BDPLT2, CD61, GP3A, GPIIIa, GT | |
| Hs.632226 | | NM_000213 | | ITGB4 | | Integrin, beta 4 | | CD104 | |
| Hs.536663 | | NM_002213 | | ITGB5 | | Integrin, beta 5 | | - | |
| Hs.521869 | | NM_000216 | | KAL1 | | Kallmann syndrome 1 sequence | | ADMLX, HH1, HHA, KAL, KALIG-1, KMS, WFDC19 | |
| Hs.270364 | | NM_005559 | | LAMA1 | | Laminin, alpha 1 | | LAMA, S-LAM-alpha | |
| Hs.200841 | | NM_000426 | | LAMA2 | | Laminin, alpha 2 | | LAMM | |
| Hs.436367 | | NM_000227 | | LAMA3 | | Laminin, alpha 3 | | BM600, E170, LAMNA, LOCS, lama3a | |
| Hs.650585 | | NM_002291 | | LAMB1 | | Laminin, beta 1 | | CLM, LIS5 | |
| Hs.497636 | | NM_000228 | | LAMB3 | | Laminin, beta 3 | | BM600-125KDA, LAM5, LAMNB1 | |
| Hs.609663 | | NM_002293 | | LAMC1 | | Laminin, gamma 1 (formerly LAMB2) | | LAMB2 | |
| Hs.83169 | | NM_002421 | | MMP1 | | Matrix metallopeptidase 1 (interstitial collagenase) | | CLG, CLGN | |
| Hs.2258 | | NM_002425 | | MMP10 | | Matrix metallopeptidase 10 (stromelysin 2) | | SL-2, STMY2 | |
| Hs.143751 | | NM_005940 | | MMP11 | | Matrix metallopeptidase 11 (stromelysin 3) | | SL-3, ST3, STMY3 | |
| Hs.709832 | | NM_002426 | | MMP12 | | Matrix metallopeptidase 12 (macrophage elastase) | | HME, ME, MME, MMP-12 | |
| Hs.2936 | | NM_002427 | | MMP13 | | Matrix metallopeptidase 13 (collagenase 3) | | CLG3, MANDP1 | |
| Hs.2399 | | NM_004995 | | MMP14 | | Matrix metallopeptidase 14 (membrane-inserted) | | MMP-14, MMP-X1, MT-MMP, MT-MMP 1, MT1-MMP, MT1MMP, MTMMP1, WNCHRS | |
| Hs.80343 | | NM_002428 | | MMP15 | | Matrix metallopeptidase 15 (membrane-inserted) | | MT2-MMP, MTMMP2, SMCP-2 | |
| Hs.492187 | | NM_005941 | | MMP16 | | Matrix metallopeptidase 16 (membrane-inserted) | | C8orf57, MMP-X2, MT-MMP2, MT-MMP3, MT3-MMP | |
| Hs.513617 | | NM_004530 | | MMP2 | | Matrix metallopeptidase 2 (gelatinase A, 72kDa gelatinase, 72kDa type IV collagenase) | | CLG4, CLG4A, MMP-II, MONA, TBE-1 | |
| Hs.375129 | | NM_002422 | | MMP3 | | Matrix metallopeptidase 3 (stromelysin 1, progelatinase) | | CHDS6, MMP-3, SL-1, STMY, STMY1, STR1 | |
| Hs.2256 | | NM_002423 | | MMP7 | | Matrix metallopeptidase 7 (matrilysin, uterine) | | MMP-7, MPSL1, PUMP-1 | |
| Hs.161839 | | NM_002424 | | MMP8 | | Matrix metallopeptidase 8 (neutrophil collagenase) | | CLG1, HNC, MMP-8, PMNL-CL | |
| Hs.297413 | | NM_004994 | | MMP9 | | Matrix metallopeptidase 9 (gelatinase B, 92kDa gelatinase, 92kDa type IV collagenase) | | CLG4B, GELB, MANDP2, MMP-9 | |
| Hs.711235 | | NM_000615 | | NCAM1 | | Neural cell adhesion molecule 1 | | CD56, MSK39, NCAM | |
| Hs.376675 | | NM_000442 | | PECAM1 | | Platelet/endothelial cell adhesion molecule | | CD31, CD31, EndoCAM, GPIIA', PECA1, PECAM-1, endoCAM | |
| Hs.82848 | | NM_000450 | | SELE | | Selectin E | | CD62E, ELAM, ELAM1, ESEL, LECAM2 | |
| Hs.728756 | | NM_000655 | | SELL | | Selectin L | | CD62L, LAM1, LECAM1, LEU8, LNHR, LSEL, LYAM1, PLNHR, TQ1 | |
| Hs.73800 | | NM_003005 | | SELP | | Selectin P (granule membrane protein 140kDa, antigen CD62) | | CD62, CD62P, GMP140, GRMP, LECAM3, PADGEM, PSEL | |
| Hs.371199 | | NM_003919 | | SGCE | | Sarcoglycan, epsilon | | DYT11, ESG | |
| Hs.111779 | | NM_003118 | | SPARC | | Secreted protein, acidic, cysteine-rich (osteonectin) | | ON | |
| Hs.185597 | | NM_003119 | | SPG7 | | Spastic paraplegia 7 (pure and complicated autosomal recessive) | | CAR, CMAR, PGN, SPG5C | |
| Hs.313 | | NM_000582 | | SPP1 | | Secreted phosphoprotein 1 | | BNSP, BSPI, ETA-1, OPN | |
| Hs.369397 | | NM_000358 | | TGFBI | | Transforming growth factor, beta-induced, 68kDa | | BIGH3, CDB1, CDG2, CDGG1, CSD, CSD1, CSD2, CSD3, EBMD, LCD1 | |
| Hs.164226 | | NM_003246 | | THBS1 | | Thrombospondin 1 | | THBS, THBS-1, TSP, TSP-1, TSP1 | |
| Hs.371147 | | NM_003247 | | THBS2 | | Thrombospondin 2 | | TSP2 | |
| Hs.658188 | | NM_007112 | | THBS3 | | Thrombospondin 3 | | TSP3 | |
| Hs.522632 | | NM_003254 | | TIMP1 | | TIMP metallopeptidase inhibitor 1 | | CLGI, EPA, EPO, HCI, TIMP | |
| Hs.633514 | | NM_003255 | | TIMP2 | | TIMP metallopeptidase inhibitor 2 | | CSC-21K, DDC8 | |
| Hs.644633 | | NM_000362 | | TIMP3 | | TIMP metallopeptidase inhibitor 3 | | HSMRK222, K222, K222TA2, SFD | |
| Hs.734766 | | NM_002160 | | TNC | | Tenascin C | | 150-225, DFNA56, GMEM, GP, HXB, JI, TN, TN-C | |
| Hs.109225 | | NM_001078 | | VCAM1 | | Vascular cell adhesion molecule 1 | | CD106, INCAM-100 | |
| Hs.643801 | | NM_004385 | | VCAN | | Versican | | CSPG2, ERVR, GHAP, PG-M, WGN, WGN1 | |
| Hs.2257 | | NM_000638 | | VTN | | Vitronectin | | V75, VN, VNT | |
| Hs.520640 | | NM_001101 | | ACTB | | Actin, beta | | BRWS1, PS1TP5BP1 | |
| Hs.534255 | | NM_004048 | | B2M | | Beta-2-microglobulin | | - | |
| Hs.544577 | | NM_002046 | | GAPDH | | Glyceraldehyde-3-phosphate dehydrogenase | | G3PD, GAPD | |
| Hs.412707 | | NM_000194 | | HPRT1 | | Hypoxanthine phosphoribosyltransferase 1 | | HGPRT, HPRT | |
| Hs.546285 | | NM_001002 | | RPLP0 | | Ribosomal protein, large, P0 | | L10E, LP0, P0, PRLP0, RPP0 | |
| N/A | | SA_00105 | | HGDC | | Human Genomic DNA Contamination | | HIGX1A | |
| N/A | | SA_00104 | | RTC | | Reverse Transcription Control | | RTC | |
| N/A | | SA_00103 | | PPC | | Positive PCR Control | | PPC | |
|  | | | | | | | | | |
| **RT² Profiler™ PCR Array Human Angiogenesis ( PAHS-024Z )** | | | | | | | | | |
| **Unigene** | **GeneBank** | | **Symbol** | | **Description** | | **Gene Name** | | |
| Hs.525622 | NM_005163 | | AKT1 | | V-akt murine thymoma viral oncogene homolog 1 | | AKT, CWS6, PKB, PKB-ALPHA, PRKBA, RAC, RAC-ALPHA | | |
| Hs.283749 | NM_001145 | | ANG | | Angiogenin, ribonuclease, RNase A family, 5 | | ALS9, HEL168, RNASE4, RNASE5 | | |
| Hs.369675 | NM_001146 | | ANGPT1 | | Angiopoietin 1 | | AGP1, AGPT, ANG1 | | |
| Hs.583870 | NM_001147 | | ANGPT2 | | Angiopoietin 2 | | AGPT2, ANG2 | | |
| Hs.9613 | NM_001039667 | | ANGPTL4 | | Angiopoietin-like 4 | | ANGPTL2, ARP4, FIAF, HARP, HFARP, NL2, PGAR, UNQ171, pp1158 | | |
| Hs.1239 | NM_001150 | | ANPEP | | Alanyl (membrane) aminopeptidase | | APN, CD13, GP150, LAP1, P150, PEPN | | |
| Hs.194654 | NM_001702 | | BAI1 | | Brain-specific angiogenesis inhibitor 1 | | GDAIF | | |
| Hs.54460 | NM_002986 | | CCL11 | | Chemokine (C-C motif) ligand 11 | | SCYA11 | | |
| Hs.303649 | NM_002982 | | CCL2 | | Chemokine (C-C motif) ligand 2 | | GDCF-2, HC11, HSMCR30, MCAF, MCP-1, MCP1, SCYA2, SMC-CF | | |
| Hs.76206 | NM_001795 | | CDH5 | | Cadherin 5, type 2 (vascular endothelium) | | 7B4, CD144 | | |
| Hs.517356 | NM_030582 | | COL18A1 | | Collagen, type XVIII, alpha 1 | | KNO, KNO1, KS | | |
| Hs.570065 | NM_000091 | | COL4A3 | | Collagen, type IV, alpha 3 (Goodpasture antigen) | | - | | |
| Hs.410037 | NM_001901 | | CTGF | | Connective tissue growth factor | | CCN2, HCS24, IGFBP8, NOV2 | | |
| Hs.708652 | NM_001511 | | CXCL1 | | Chemokine (C-X-C motif) ligand 1 (melanoma growth stimulating activity, alpha) | | FSP, GRO1, GROa, MGSA, MGSA-a, NAP-3, SCYB1 | | |
| Hs.632586 | NM_001565 | | CXCL10 | | Chemokine (C-X-C motif) ligand 10 | | C7, IFI10, INP10, IP-10, SCYB10, crg-2, gIP-10, mob-1 | | |
| Hs.89714 | NM_002994 | | CXCL5 | | Chemokine (C-X-C motif) ligand 5 | | ENA-78, SCYB5 | | |
| Hs.164021 | NM_002993 | | CXCL6 | | Chemokine (C-X-C motif) ligand 6 (granulocyte chemotactic protein 2) | | CKA-3, GCP-2, GCP2, SCYB6 | | |
| Hs.77367 | NM_002416 | | CXCL9 | | Chemokine (C-X-C motif) ligand 9 | | CMK, Humig, MIG, SCYB9, crg-10 | | |
| Hs.713645 | NM_001955 | | EDN1 | | Endothelin 1 | | ET1, HDLCQ7, PPET1 | | |
| Hs.516664 | NM_182685 | | EFNA1 | | Ephrin-A1 | | B61, ECKLG, EFL1, EPLG1, LERK-1, LERK1, TNFAIP4 | | |
| Hs.149239 | NM_004093 | | EFNB2 | | Ephrin-B2 | | EPLG5, HTKL, Htk-L, LERK5 | | |
| Hs.419815 | NM_001963 | | EGF | | Epidermal growth factor | | HOMG4, URG | | |
| Hs.76753 | NM_000118 | | ENG | | Endoglin | | END, HHT1, ORW1 | | |
| Hs.437008 | NM_004444 | | EPHB4 | | EPH receptor B4 | | HTK, MYK1, TYRO11 | | |
| Hs.446352 | NM_004448 | | ERBB2 | | V-erb-b2 erythroblastic leukemia viral oncogene homolog 2, neuro/glioblastoma derived oncogene homolog (avian) | | CD340, HER-2, HER-2, neu, HER2, MLN 19, NEU, NGL, TKR1 | | |
| Hs.62192 | NM_001993 | | F3 | | Coagulation factor III (thromboplastin, tissue factor) | | CD142, TF, TFA | | |
| Hs.483635 | NM_000800 | | FGF1 | | Fibroblast growth factor 1 (acidic) | | AFGF, ECGF, ECGF-beta, ECGFA, ECGFB, FGF-1, FGF-alpha, FGFA, GLIO703, HBGF-1, HBGF1 | | |
| Hs.284244 | NM_002006 | | FGF2 | | Fibroblast growth factor 2 (basic) | | BFGF, FGF-2, FGFB, HBGF-2 | | |
| Hs.1420 | NM_000142 | | FGFR3 | | Fibroblast growth factor receptor 3 | | ACH, CD333, CEK2, HSFGFR3EX, JTK4 | | |
| Hs.11392 | NM_004469 | | FIGF | | C-fos induced growth factor (vascular endothelial growth factor D) | | VEGF-D, VEGFD | | |
| Hs.594454 | NM_002019 | | FLT1 | | Fms-related tyrosine kinase 1 (vascular endothelial growth factor/vascular permeability factor receptor) | | FLT, FLT-1, VEGFR-1, VEGFR1 | | |
| Hs.203717 | NM_002026 | | FN1 | | Fibronectin 1 | | CIG, ED-B, FINC, FN, FNZ, GFND, GFND2, LETS, MSF | | |
| Hs.396530 | NM_000601 | | HGF | | Hepatocyte growth factor (hepapoietin A; scatter factor) | | DFNB39, F-TCF, HGFB, HPTA, SF | | |
| Hs.719495 | NM_001530 | | HIF1A | | Hypoxia inducible factor 1, alpha subunit (basic helix-loop-helix transcription factor) | | HIF-1A, HIF-1alpha, HIF1, HIF1-ALPHA, MOP1, PASD8, bHLHe78 | | |
| Hs.44227 | NM_006665 | | HPSE | | Heparanase | | HPA, HPA1, HPR1, HPSE1, HSE1 | | |
| Hs.504609 | NM_002165 | | ID1 | | Inhibitor of DNA binding 1, dominant negative helix-loop-helix protein | | ID, bHLHb24 | | |
| Hs.37026 | NM_024013 | | IFNA1 | | Interferon, alpha 1 | | IFL, IFN, IFN-ALPHA, IFN-alphaD, IFNA13, IFNA@ | | |
| Hs.856 | NM_000619 | | IFNG | | Interferon, gamma | | IFG, IFI | | |
| Hs.160562 | NM_000618 | | IGF1 | | Insulin-like growth factor 1 (somatomedin C) | | IGF-I, IGF1A, IGFI | | |
| Hs.126256 | NM_000576 | | IL1B | | Interleukin 1, beta | | IL-1, IL1-BETA, IL1F2 | | |
| Hs.654458 | NM_000600 | | IL6 | | Interleukin 6 (interferon, beta 2) | | BSF2, HGF, HSF, IFNB2, IL-6 | | |
| Hs.624 | NM_000584 | | IL8 | | Interleukin 8 | | CXCL8, GCP-1, GCP1, LECT, LUCT, LYNAP, MDNCF, MONAP, NAF, NAP-1, NAP1 | | |
| Hs.436873 | NM_002210 | | ITGAV | | Integrin, alpha V (vitronectin receptor, alpha polypeptide, antigen CD51) | | CD51, MSK8, VNRA, VTNR | | |
| Hs.218040 | NM_000212 | | ITGB3 | | Integrin, beta 3 (platelet glycoprotein IIIa, antigen CD61) | | BDPLT16, BDPLT2, CD61, GP3A, GPIIIa, GT | | |
| Hs.626544 | NM_000214 | | JAG1 | | Jagged 1 | | AGS, AHD, AWS, CD339, HJ1, JAGL1 | | |
| Hs.479756 | NM_002253 | | KDR | | Kinase insert domain receptor (a type III receptor tyrosine kinase) | | CD309, FLK1, VEGFR, VEGFR2 | | |
| Hs.421391 | NM_007015 | | LECT1 | | Leukocyte cell derived chemotaxin 1 | | BRICD3, CHM-I, CHM1, MYETS1 | | |
| Hs.194236 | NM_000230 | | LEP | | Leptin | | LEPD, OB, OBS | | |
| Hs.82045 | NM_002391 | | MDK | | Midkine (neurite growth-promoting factor 2) | | ARAP, MK, NEGF2 | | |
| Hs.2399 | NM_004995 | | MMP14 | | Matrix metallopeptidase 14 (membrane-inserted) | | MMP-14, MMP-X1, MT-MMP, MT-MMP 1, MT1-MMP, MT1MMP, MTMMP1, WNCHRS | | |
| Hs.513617 | NM_004530 | | MMP2 | | Matrix metallopeptidase 2 (gelatinase A, 72kDa gelatinase, 72kDa type IV collagenase) | | CLG4, CLG4A, MMP-II, MONA, TBE-1 | | |
| Hs.297413 | NM_004994 | | MMP9 | | Matrix metallopeptidase 9 (gelatinase B, 92kDa gelatinase, 92kDa type IV collagenase) | | CLG4B, GELB, MANDP2, MMP-9 | | |
| Hs.647092 | NM_000603 | | NOS3 | | Nitric oxide synthase 3 (endothelial cell) | | ECNOS, eNOS | | |
| Hs.436100 | NM_004557 | | NOTCH4 | | Notch 4 | | INT3, NOTCH3 | | |
| Hs.653996 | NM_003873 | | NRP1 | | Neuropilin 1 | | BDCA4, CD304, NP1, NRP, VEGF165R | | |
| Hs.471200 | NM_003872 | | NRP2 | | Neuropilin 2 | | NP2, NPN2, PRO2714, VEGF165R2 | | |
| Hs.535898 | NM_002607 | | PDGFA | | Platelet-derived growth factor alpha polypeptide | | PDGF-A, PDGF1 | | |
| Hs.376675 | NM_000442 | | PECAM1 | | Platelet/endothelial cell adhesion molecule | | CD31, CD31, EndoCAM, GPIIA', PECA1, PECAM-1, endoCAM | | |
| Hs.81564 | NM_002619 | | PF4 | | Platelet factor 4 | | CXCL4, PF-4, SCYB4 | | |
| Hs.252820 | NM_002632 | | PGF | | Placental growth factor | | D12S1900, PGFL, PLGF, PlGF-2, SHGC-10760 | | |
| Hs.77274 | NM_002658 | | PLAU | | Plasminogen activator, urokinase | | ATF, BDPLT5, QPD, UPA, URK, u-PA | | |
| Hs.143436 | NM_000301 | | PLG | | Plasminogen | | - | | |
| Hs.528665 | NM_021935 | | PROK2 | | Prokineticin 2 | | BV8, HH4, KAL4, MIT1, PK2 | | |
| Hs.201978 | NM_000962 | | PTGS1 | | Prostaglandin-endoperoxide synthase 1 (prostaglandin G/H synthase and cyclooxygenase) | | COX1, COX3, PCOX1, PES-1, PGG, HS, PGHS-1, PGHS1, PHS1, PTGHS | | |
| Hs.154210 | NM_001400 | | S1PR1 | | Sphingosine-1-phosphate receptor 1 | | CD363, CHEDG1, D1S3362, ECGF1, EDG-1, EDG1, S1P1 | | |
| Hs.713079 | NM_000602 | | SERPINE1 | | Serpin peptidase inhibitor, clade E (nexin, plasminogen activator inhibitor type 1), member 1 | | PAI, PAI-1, PAI1, PLANH1 | | |
| Hs.532768 | NM_002615 | | SERPINF1 | | Serpin peptidase inhibitor, clade F (alpha-2 antiplasmin, pigment epithelium derived factor), member 1 | | EPC-1, OI12, OI6, PEDF | | |
| Hs.68061 | NM_021972 | | SPHK1 | | Sphingosine kinase 1 | | SPHK | | |
| Hs.89640 | NM_000459 | | TEK | | TEK tyrosine kinase, endothelial | | CD202B, TIE-2, TIE2, VMCM, VMCM1 | | |
| Hs.628298 | NM_003236 | | TGFA | | Transforming growth factor, alpha | | TFGA | | |
| Hs.645227 | NM_000660 | | TGFB1 | | Transforming growth factor, beta 1 | | CED, DPD1, LAP, TGFB, TGFbeta | | |
| Hs.133379 | NM_003238 | | TGFB2 | | Transforming growth factor, beta 2 | | LDS4, TGF-beta2 | | |
| Hs.494622 | NM_004612 | | TGFBR1 | | Transforming growth factor, beta receptor 1 | | AAT5, ACVRLK4, ALK-5, ALK5, LDS1A, LDS2A, MSSE, SKR4, TGFR-1 | | |
| Hs.164226 | NM_003246 | | THBS1 | | Thrombospondin 1 | | THBS, THBS-1, TSP, TSP-1, TSP1 | | |
| Hs.371147 | NM_003247 | | THBS2 | | Thrombospondin 2 | | TSP2 | | |
| Hs.78824 | NM_005424 | | TIE1 | | Tyrosine kinase with immunoglobulin-like and EGF-like domains 1 | | JTK14, TIE | | |
| Hs.522632 | NM_003254 | | TIMP1 | | TIMP metallopeptidase inhibitor 1 | | CLGI, EPA, EPO, HCI, TIMP | | |
| Hs.633514 | NM_003255 | | TIMP2 | | TIMP metallopeptidase inhibitor 2 | | CSC-21K, DDC8 | | |
| Hs.644633 | NM_000362 | | TIMP3 | | TIMP metallopeptidase inhibitor 3 | | HSMRK222, K222, K222TA2, SFD | | |
| Hs.241570 | NM_000594 | | TNF | | Tumor necrosis factor | | DIF, TNF-alpha, TNFA, TNFSF2 | | |
| Hs.730607 | NM_001953 | | TYMP | | Thymidine phosphorylase | | ECGF, ECGF1, MEDPS1, MNGIE, MTDPS1, PDECGF, TP, hPD-ECGF | | |
| Hs.73793 | NM_003376 | | VEGFA | | Vascular endothelial growth factor A | | MVCD1, VEGF, VPF | | |
| Hs.732095 | NM_003377 | | VEGFB | | Vascular endothelial growth factor B | | VEGFL, VRF | | |
| Hs.435215 | NM_005429 | | VEGFC | | Vascular endothelial growth factor C | | Flt4-L, VRP | | |
| Hs.520640 | NM_001101 | | ACTB | | Actin, beta | | BRWS1, PS1TP5BP1 | | |
| Hs.534255 | NM_004048 | | B2M | | Beta-2-microglobulin | | - | | |
| Hs.544577 | NM_002046 | | GAPDH | | Glyceraldehyde-3-phosphate dehydrogenase | | G3PD, GAPD | | |
| Hs.412707 | NM_000194 | | HPRT1 | | Hypoxanthine phosphoribosyltransferase 1 | | HGPRT, HPRT | | |
| Hs.546285 | NM_001002 | | RPLP0 | | Ribosomal protein, large, P0 | | L10E, LP0, P0, PRLP0, RPP0 | | |
| N/A | SA_00105 | | HGDC | | Human Genomic DNA Contamination | | HIGX1A | | |
| N/A | SA_00104 | | RTC | | Reverse Transcription Control | | RTC | | |
| N/A | SA_00104 | | RTC | | Reverse Transcription Control | | RTC | | |
| N/A | SA_00104 | | RTC | | Reverse Transcription Control | | RTC | | |
| N/A | SA_00103 | | PPC | | Positive PCR Control | | PPC | | |
| N/A | SA_00103 | | PPC | | Positive PCR Control | | PPC | | |
| N/A | SA_00103 | | PPC | | Positive PCR Control | | PPC | | |
|  |  | |  | |  | |  | |  |
| **qBiomarker iPSC PCR Array Cardiomyocytes differentiation**  **(IPHS-102A)** | | | | | | | | |  |
|  | | | | | | | | |  |
| **Unigene** | **GeneBank** | | **Symbol** | | **Description** | | **Gene Name** | |  |
| Hs.498178 | NM_001103 | | ACTN2 | | Actinin, alpha 2 | | CMD1AA | |  |
| Hs.719946 | NM_000257 | | MYH7 | | Myosin, heavy chain 7, cardiac muscle, beta | | CMD1S, CMH1, MPD1, MYHCB, SPMD, SPMM | |  |
| Hs.744988 | NM_021097 | | SLC8A1 | | Solute carrier family 8 (sodium/calcium exchanger), member 1 | | NCX1 | |  |
| Hs.99913 | NM_000684 | | ADRB1 | | Adrenergic, beta-1-, receptor | | ADRB1R, B1AR, BETA1AR, RHR | |  |
| Hs.75535 | NM_000432 | | MYL2 | | Myosin, light chain 2, regulatory, cardiac, slow | | CMH10, MLC2 | |  |
| Hs.709179 | NM_000363 | | TNNI3 | | Troponin I type 3 (cardiac) | | CMD1FF, CMD2A, CMH7, RCM1, TNNC1, cTnI | |  |
| Hs.334347 | NM_001824 | | CKM | | Creatine kinase, muscle | | CKMM, M-CK | |  |
| Hs.517939 | NM_000258 | | MYL3 | | Myosin, light chain 3, alkali; ventricular, skeletal, slow | | CMH8, MLC1SB, MLC1V, VLC1 | |  |
| Hs.533613 | NM_000364 | | TNNT2 | | Troponin T type 2 (cardiac) | | CMH2, CMPD2, LVNC6, RCM3, TnTC, cTnT | |  |
| Hs.594952 | NM_001927 | | DES | | Desmin | | CSM1, CSM2, LGMD2R | |  |
| Hs.75636 | NM_021223 | | MYL7 | | Myosin, light chain 7, regulatory | | MYL2A, MYLC2A | |  |
| Hs.591847 | NM_000662 | | NAT1 | | N-acetyltransferase 1 (arylamine N-acetyltransferase) | | AAC1, MNAT, NAT-1, NATI | |  |
| Hs.243987 | NM_002052 | | GATA4 | | GATA binding protein 4 | | ASD2, TACHD, VSD1 | |  |
| Hs.54473 | NM_004387 | | NKX2-5 | | NK2 homeobox 5 | | CHNG5, CSX, CSX1, HLHS2, NKX2.5, NKX2E, NKX4-1, VSD3 | |  |
| Hs.544577 | NM_002046 | | GAPDH | | Glyceraldehyde-3-phosphate dehydrogenase | | G3PD, GAPD | |  |
| Hs.388245 | NM_021973 | | HAND2 | | Heart and neural crest derivatives expressed 2 | | DHAND2, Hed, Thing2, bHLHa26, dHand | |  |
| Hs.75640 | NM_006172 | | NPPA | | Natriuretic peptide A | | ANF, ANP, ATFB6, CDD-ANF, PND | |  |
| N/A | SA_00105 | | HGDC | | Human Genomic DNA Contamination | | HIGX1A | |  |
| Hs.95162 | NM_000218 | | KCNQ1 | | Potassium voltage-gated channel, KQT-like subfamily, member 1 | | ATFB1, ATFB3, JLNS1, KCNA8, KCNA9, KVLQT1, Kv1.9, Kv7.1, LQT, LQT1, RWS, SQT2, WRS | |  |
| Hs.170839 | NM_002667 | | PLN | | Phospholamban | | CMD1P, CMH18, PLB | |  |
| N/A | SA_00104 | | RTC | | Reverse Transcription Control | | RTC | |  |
| Hs.517586 | NM_005368 | | MB | | Myoglobin | | PVALB, myoglobgin | |  |
| Hs.738571 | NM_001035 | | RYR2 | | Ryanodine receptor 2 (cardiac) | | ARVC2, ARVD2, RYR-2, RyR, VTSIP | |  |
| N/A | SA_00103 | | PPC | | Positive PCR Control | | PPC | |  |

## Supplementary Figures


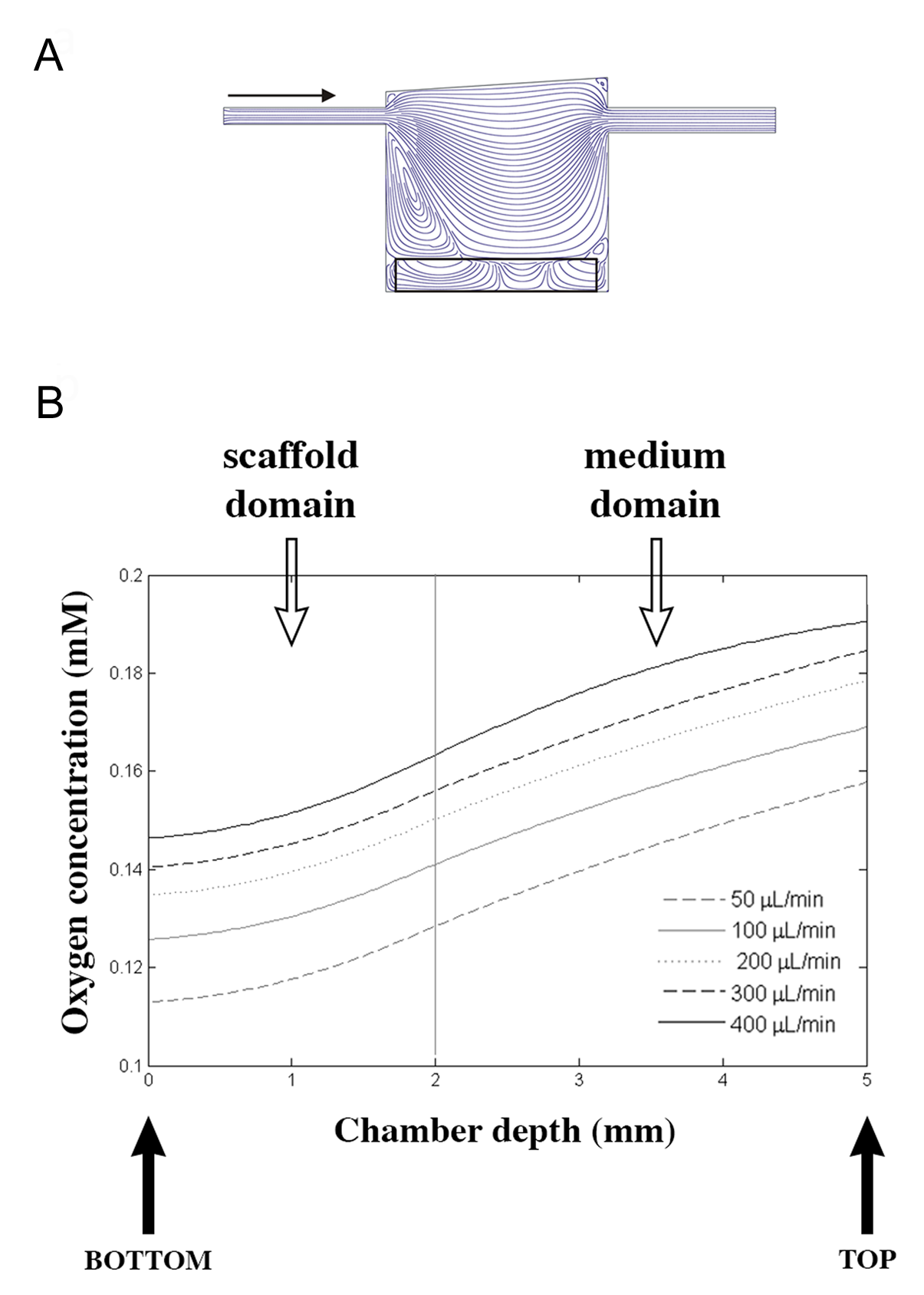


**Supplementary Figure 1.** Section of oxygen concentration in the bioreactor chamber and scaffold domains for different flow rates (**A**). Streamlines of fluid flow in the bioreactor chamber (**B**) when the inner flow rate is set at a value of 200 μL/min. The scaffold placed to the bottom of the perfusion chamber is 2 mm in height and 12 mm diameter.


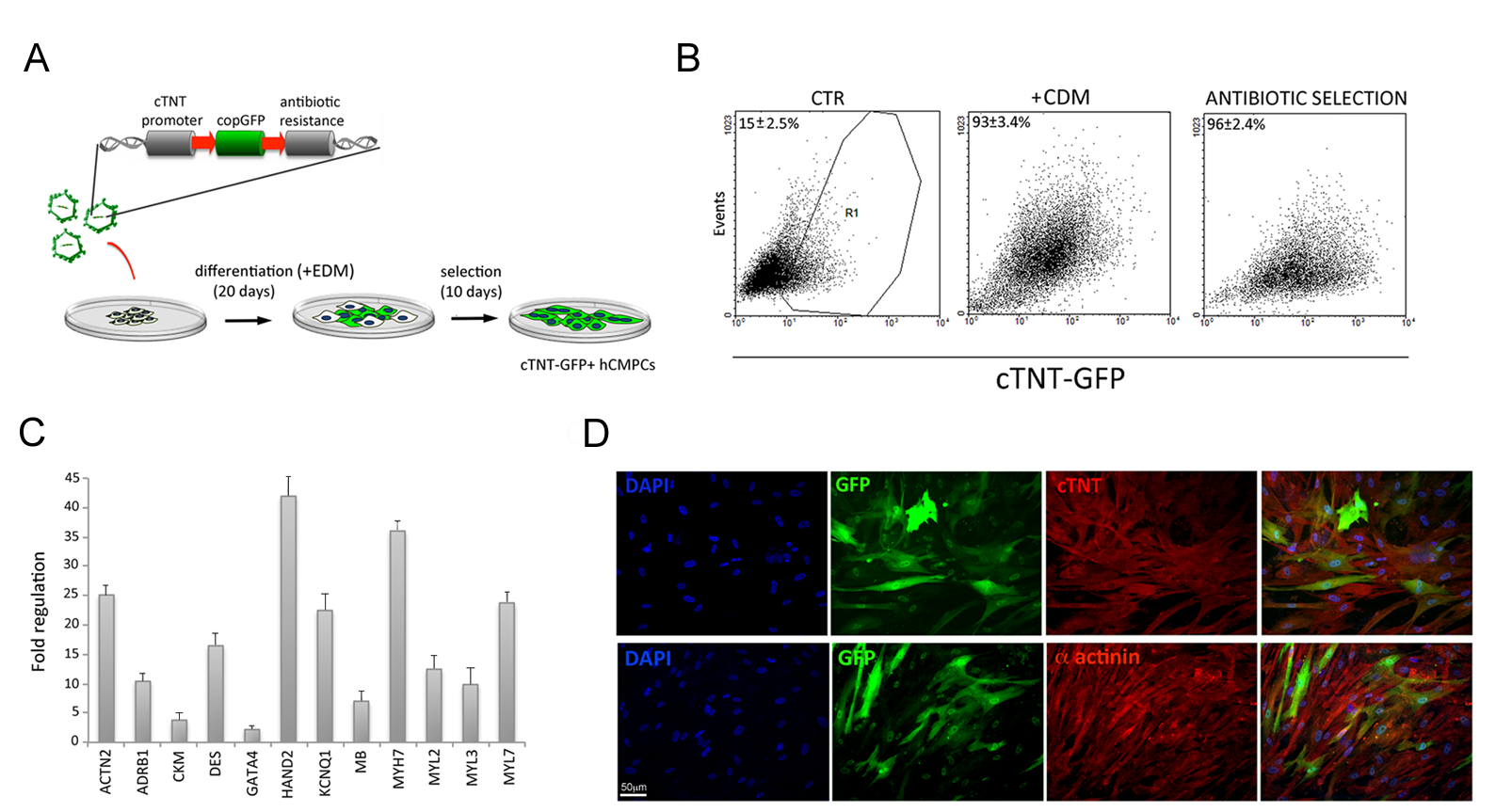


**Supplementary Figure 2.** Human cardiac progenitor cells acquire cardiomyocyte phenotype *in vitro*. The ability of human cardiac progenitors (hCMPCs) can be confirmed by transducing the cells with a viral vector codifying for GFP reporter gene under cardiac Troponin T (cTNT) promoter (**A**). The vector, also encoding for zeocin resistance, allows for the enrichment of differentiated cells. GFP expression has been quantified by FACS analysis in cTNT-GFP hCMPCs grown in hCMPCs basal medium (CTR) or stimulated with cardiac differentiation medium (CDM) for 20 days (**B**), before and after antibiotic selection. After 20 days of stimulation in CDM and antibiotic selection, quantitative PCR showed the upregulation of several cardiac specific genes as compared to cells grown in hCMPCs basal medium for 20 days (**C,** refer to TABLE 1 for the complete list of genes). Cell differentiation has also been confirmed by immunofluorescence showing the great majority of the cells co-expressing GFP (green) and cTNT or alpha actinin (**D**).

**
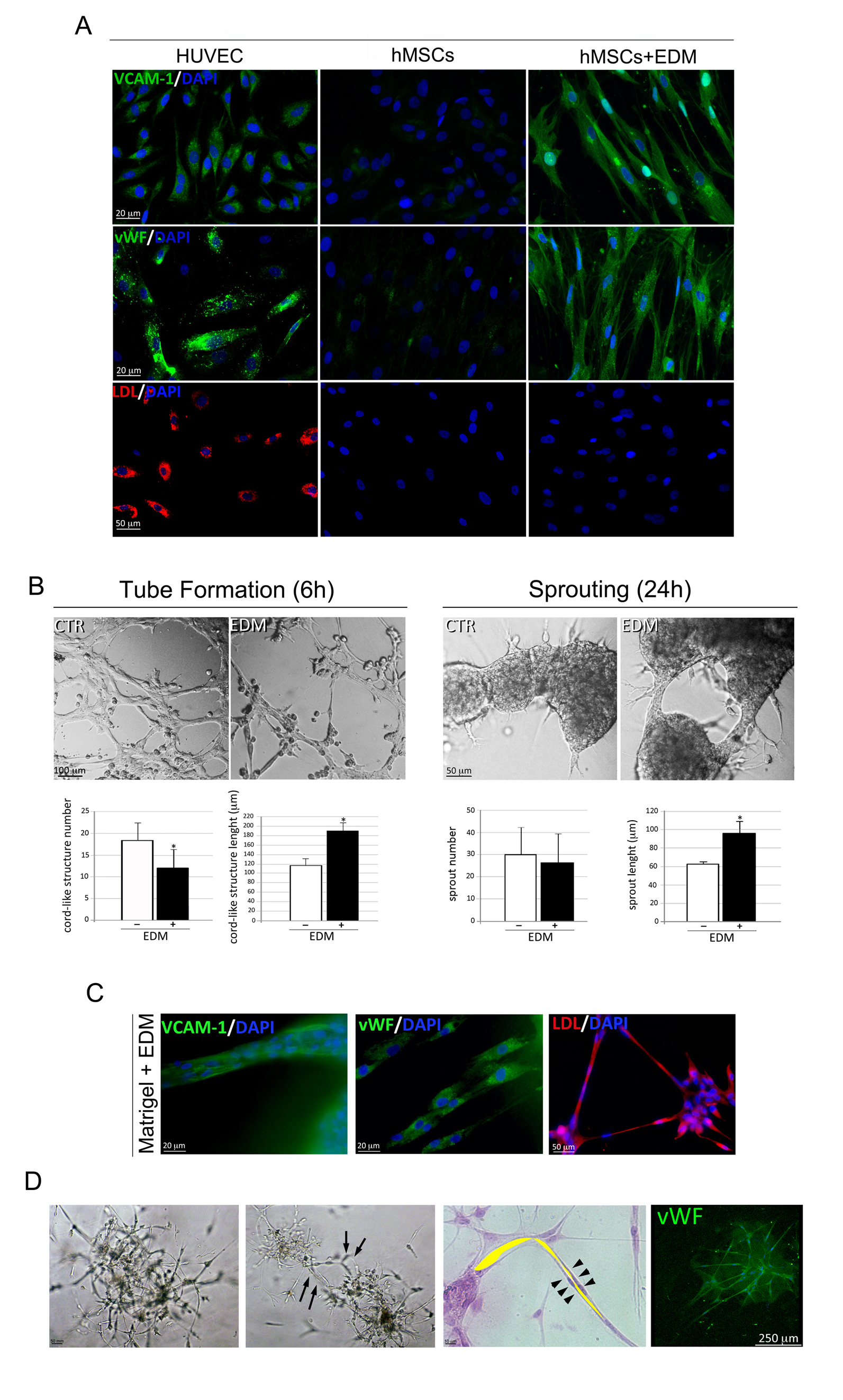
**

**Supplementary Figure 3.** Human mesenchymal stem cells acquire the endothelial phenotype and generate interconnected vessel-like structures when stimulated with Endothelial differentiation medium (EDM) in Matrigel™. Human MSCs was able to express endothelial markers like VCAM-1 and vWF, but not to uptake Ac-LDL upon EDM stimulation for 7 days (**A**) in 2D standard culture (TCPS). HUVEC cells, cultured under the same conditions, were used as positive control. Human MSCs cultured in Matrigel™ 3D environment migrated and branched to form a well-organized network of tubules (**B**) resembling vascular structures after 6 hours (left panels). The number of vessel-like structures appeared to be lower while their length was increased upon EDM stimulation. Twenty-four hour later, this complex branching network collapsed, while small sprouts, suggestive of capillary structures, outgrew (right panels). The number of sprouting capillaries was not affected by EDM stimulation while a significant increment in their length was observed. In 3D culture conditions, cells acquired the ability to uptake acetylated LDL and lined to form vessel-like structures (**C**). Four day later, (**D**) radially anastomosing and interconnected capillary sprouts (black arrows, phase contrast) were visible and formed a complex network of luminized structures (pseudo-colored in yellow in H&E staining) with lining endothelial cells (black arrowheads) expressing vWF.

1. **References^[[1]](#footnote-1)^**

Abramoff, M. D., Magalhaes, P. J., and Ram, S. J. (2004). Image Processing with ImageJ. *Biophotonics International* 11:36-42.

Brannon-Peppas, L., and Peppas, N.A. (1990). Dynamic and equilibrium swelling behaviour of pH-sensitive hydrogels containing 2-hydroxyethyl methacrylate. *Biomaterials* 11, 635–644.

Forte, G., Pagliari, S., Ebara, U., Uto, K., Tam, J. K., Romanazzo, S., et al. (2012). Substrate stiffness modulates neonatal cardiomyocyte maturation in vitro. *Tissue Eng. Part. A* 18, 1837-1848. doi: 10.1089/ten.TEA.2011.0707.

Lien, S. M., Ko, L. Y., and Huang, T. J. (2009). Effect of pore size and ECM secretion and cell growth in gelatin scaffold for articular cartilage tissue engineering. *Acta Biomater.* 5, 670-679. doi: 10.1016/j.actbio.2008.09.020.

Martucci, J. F., Ruseckaite, R. A, and Vàzquez, A. (2006). Creep of glutaraldehyde-crosslinked gelatin films. *Materials Science & Engineering A* 435-436, 681-686. doi: 10.1016/j.msea.2006.07.097.

Mwangi, J. W., and Ofner, C. M. 3rd. (2004). Crosslinked gelatin matrices: release of a random coil macromolecular solute. *Int. J. Pharm.* 278, 319-327.

Spinelli, A., Vinci, B., Tirella, A., Matteucci, M., Gargani, L., Ahluwalia, A., et al. (2012). Realization of a poro-elastic ultrasound replica of pulmonary tissue. *Biomatter* 2:37-42. doi: 10.4161/biom.19835.

1. Provide the doi when available, and ALL complete author names. [↑](#footnote-ref-1)
